# A Family of Sequential Item Response Models for Multiple-Choice, Multiple-Attempt Test Items

**DOI:** 10.1017/psy.2024.18

**Published:** 2025-01-03

**Authors:** Yikai Lu, Jim Fowler, Ying Cheng

**Affiliations:** 1 Department of Psychology, University of Notre Dame, Notre Dame, IN, USA; 2 Department of Mathematics, The Ohio State University, Columbus, OH, USA

**Keywords:** answer-until-correct, multiple attempts, multiple choice item, sequential item response theory

## Abstract

We consider a test which allows students to attempt a multiple-choice question multiple times (i.e., multiple attempts). The most extreme form of multiple attempts is the answer-until-correct (AUC) procedure. Previous research has demonstrated that multiple-attempt procedures such as AUC could potentially enhance learning and increase reliability. However, for multiple-choice items, guessing is still non-ignorable. Traditional models of sequential item response theory (SIRT) could model multiple-attempt procedures but fail to take guessing into account. The purpose of this study is to propose SIRT models for multiple-choice, multiple-attempt items (SIRT-MM). First, we defined a family of SIRT-MM models to account for the idiosyncrasies of items, answer options, and examinee behavior. We also explained how these models could improve person parameter estimates by taking into account partial (mis)information of examinees. Second, we conducted model comparisons between the SIRT-MM models, the graded response model, and the nominal response model. Third, we discussed how the item and person parameters can be estimated, and evaluated item and person parameter recovery of SIRT-MM models under different conditions through a simulation study. Finally, we applied the SIRT-MM models to a real dataset and demonstrated their utility through model selection, person parameter recovery, and information functions.

## 1 Introduction

Many tests employ two types of items: constructed-response items and multiple-choice items (Kastner & Stangla, 2011; Lukhele et al., 1994). Constructed response questions require examinees to create their own answers, which can take many forms, including short text responses, an essay, a diagram, an explanation of a procedure, or the step-by-step solution of a mathematical problem (Kastner & Stangla, 2011; Lukhele et al., 1994). Multiple-choice is an item format widely used in testing due to its simplicity of scoring, which consists of answer choices (or alternatives) and in many cases one of them is the correct choice.

Scoring of multiple-choice items can heavily depend on the state of an examinee. The simplest example would be a completely ignorant examinee, who could guess the correct answer choice and possibly receive credit by chance. Specifically, letting *K* be the total number of answer choices and assuming the examinee does not have any knowledge of a test item, the probability of guessing the correct response, or the expected score when 0/1 (correct-or-incorrect) scoring is used, is 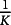 . We refer to this condition as complete ignorance. An examinee who could eliminate some distractors, or wrong answer choices, will have a higher chance to earn a point. For example, the expected score will be 0.5 when an examinee could eliminate 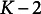 distractors and leave two possible choices including the correct answer choice. Davis (1964) referred to this condition, where an examinee guesses among some, not all, correct and incorrect choices, as partial information (Frary, 1980). On the other hand, Davis (1964) referred to a condition where an examinee is misinformed and eliminates the correct choice, as misinformation (Frary, 1980). The amount of misinformation varies depending on how many choices, including the correct choice, are believed to be incorrect. For instance, if an examinee believes that the correct answer choice and 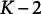 distractors are incorrect, he or she would select the remaining distractor, getting 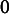 point by 0/1 scoring. On the other hand, if an examinee believes that the actual correct choice is wrong but all the distractors are correct, he or she would select one of the distractors, getting 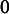 point by 0/1 scoring as well. The former condition is referred to as partial misinformation and the latter condition is referred to as complete misinformation (Frary, 1980). Intuitively, complete misinformation should be penalized more than partial misinformation. Nevertheless, such conditions are handled differently depending on scoring methods. For example, the simple 0/1 scoring method will treat these two misinformation conditions equally, as both examinees would select one of the distractors. However, an answer-until-correct (AUC) procedure, which is a popular multiple-attempt procedure that lets an examinee continue to select answer options until the correct option is picked, tends to give higher expected item scores with lower levels of misinformation (Frary, 1980, 1989; Kane & Moloney, 1978). Frary (1980) gave a good summary of how these two misinformation conditions are handled in various scoring methods including a multiple-attempt procedure, which is the focus of this article.

As a multiple-attempt procedure, AUC has been reported to have various advantages including: (1) AUC can lead to higher reliability than 0/1 scoring by taking into account different levels of examinees’ partial (mis)information (Gilman & Ferry, 1972; Hanna, 1975; Slepkov & Godfrey, 2019), (2) AUC could enhance learning by providing immediate corrective feedback between attempts (Epstein et al., 2001), and (3) AUC is strongly preferred by examinees over only one attempt being allowed (DiBattista et al., 2009). Importantly, Epstein et al. (2001) found that their AUC procedure significantly enhances the retention of material from earlier exams. Specifically, in the final exam, students who had previously experienced the AUC approach were twice as likely to answer previously incorrect questions correctly compared to those who had used Scantron forms (Epstein et al., 2001).

Item scoring for a multiple-attempt procedure, including the AUC procedure, can be very simple: 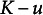 where *u* is the number of attempts an examinee makes. In this way, we can retrieve the levels of partial misinformation by recording the number of attempts. For example, a completely misinformed examinee would continuously select distractors until the last attempt, resulting in zero points, whereas a partially misinformed examinee who believes that one distractor is correct and is unsure about the other choices, would select that distractor at the first attempt and guess from the second attempt on. Thus, the expected score of a partially misinformed examinee would be higher than that of a completely misinformed examinee. A multiple-attempt procedure can also take into account the different levels of partial information. For example, if a partially informed examinee could eliminate a number of distractors and leave *s* remaining choices, they are guaranteed to have a score of 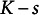 or better. Moreover, different item scoring schemes are possible. Slepkov and Godfrey (2019) conducted analyses of the reliability of several multiple-attempt tests using different item scoring schemes. In Slepkov and Godfrey (2019), the most popular scoring scheme is one that grants full credit if the first attempt was successful, half credit if the second attempt is successful, one-tenth credit if the third attempt was successful, and no credit otherwise.

These scoring schemes are based on classical test theory. Classical test theory is a class of measurement models that are based on the total sum of item scores, and typically each item is scored by the 0/1 scoring. When such scoring schemes for a multiple-attempt procedure are used, we calculate the total sum of item scores as an estimate of the ability of an examinee. Another approach to model the ability of an examinee is to use item response theory (IRT; De Ayala, 2009). Tutz (1990) proposed sequential item response (SIRT) models, which are motivated by “a genuine stepwise approach” to emphasize its difference from the partial credit model. Unlike the latter, SIRT models can model a person’s consecutive attempts at an item, such as a test of psychomotor skills. One advantage of SIRT models is that item parameters specific for future attempts do not affect ability estimation at the earlier attempts (Tutz, 1990). Thus, SIRT models could be used for modeling a multiple-attempt procedure that allows an unlimited or limited number of attempts.

More specifically, following the notations in Culpepper (2014), SIRT models could be formulated as follows. Suppose a test item has a multiple-attempt procedure that allows an examinee to submit answers until they reach the correct answer. Let 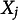 be a random variable representing the number of attempts an examinee needed to submit a correct answer on item *j* and 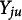 represents a Bernoulli random variable of whether the examinee submitted a correct or incorrect response on attempt *u*. In SIRT, 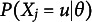 where 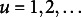 could be constructed by assuming 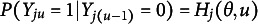 and 
(1)

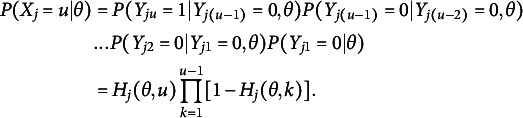



We often assume that 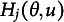 is a function of item parameters, 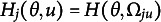 , where 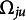 is item parameters for item *j* at attempt *u*. By assuming 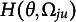 to be a Rasch model, 
(2)

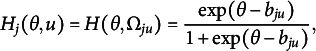



where 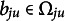 , we get the Rasch sequential item response model, which was proposed by Tutz (1990).

However, one problem in using the Rasch sequential item response model for multiple-choice, multiple-attempt test items is that it does not take into account guessing at each attempt, which can be non-trivial especially at later attempts when some answer options have already been eliminated. While existing SIRT models are suitable for a multiple-attempt procedure with constructed responses, an appropriate model is yet to be proposed for a multiple-attempt procedure with multiple-choice items. Thus, the goal of this study is to formally propose new SIRT models, which we call the “SIRT-MM” models (MM stands for multiple-choice, multiple-mttempt), to effectively score multiple-attempt responses for multiple-choice test items. This will be achieved by taking into account the structure of a multiple-choice test item, especially considering the homogeneity or heterogeneity of distractors and the process of elimination of answer choices after reattempts. As a result, we will address the issue of guessing at each attempt. Subsequently, we will also (1) evaluate parameter recovery under various test length and sample size conditions, (2) compare SIRT-MM models with competing models such as the graded response model and the nominal response model for multiple attempts data, and (3) demonstrate the usage of SIRT-MM models using real data.

## 2 Methods

### 2.1 The basic SIRT-MM model

In this section, we will suppress the subscript *j* denoting individual items for simplicity (e.g., denoting 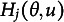 as 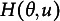 ). Theoretically, to model items using SIRT models, we can design any function for 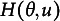 , and thus an infinite number of the variants of SIRT models could be created. In our context, we need to consider a 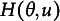 suitable for multiple-choice test items. We begin by considering the structure of a multiple-choice item. Suppose a multiple-choice item has *K* choices, including one correct choice and 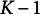 distractors, and its choice set as 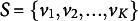 , which is a set of all *K* answer choices of a multiple-choice item. We allow *K* attempts because any examinee could reach a correct choice by the *K*th attempt by eliminating all the distractors. Technically speaking, we only need 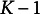 attempts. However, for the sake of the clarity of our models, we will include the *K*th attempt as a response category to differentiate whether the 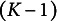 th attempt is successful or not.

We model multiple-choice, multiple-attempt test items following the discrete choice theory (Agresti, 2013; Ben-Akiva, 1985; Benson et al., 2016; Luce, 1959). The fundamental principle of discrete choice analysis is utility maximization, which assumes that a decision maker selects the option or alternative with the highest utility among all available alternatives at the time (Ben-Akiva, 1985). In the testing context, each answer option of a multiple-choice test item is considered an alternative, and an examinee’s perceived correctness of an option is its utility. Utility maximization means an examinee would always try to choose an answer option with the highest perceived correctness.

However, the deterministic view of the choice theory has a limitation in modeling examinees’ behavior using a single latent variable 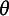 , because 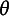 cannot fully explain the variations of item responses and there always is some randomness not captured. Therefore, we will adopt the probabilistic choice theory for modeling examinees’ behavior.

In probabilistic choice theory, the “choice axiom” (Ben-Akiva, 1985; Luce, 1959) states that the probability of choosing any answer choice *v* from the choice set *S* would satisfy 
(3)

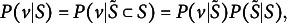



where 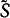 is any subset of *S* and 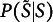 is the probability of choosing an answer choice in 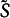 . The choice axiom suggests that if some distractors are removed from the choice set, the relative probabilities for the remaining options are unchanged (Ben-Akiva, 1985). The “choice axiom” implies *independence from irrelevant alternatives* (IIA; Luce, 1959): 
(4)

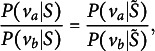



which suggests that the odds of choosing 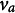 over 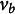 do not depend on the other options in the choice set (Agresti, 2013).

The IIA assumption is widely used and discussed in statistics literature (Agresti, 2013; Benson et al., 2016), such as in multinomial logit models, e.g., multinomial logistic regression (Agresti, 2013; Ben-Akiva, 1985), and other “divide-by-total” models (Thissen & Steinberg, 1986), e.g., (generalized) partial credit model (Masters, 1982; Muraki, 1992) and nominal response model (Bock, 1972). In fact, the divide-by-total models can be derived under the IIA assumption. In other words, when IIA holds, a utility model of 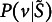 for any 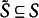 will be 
(5)

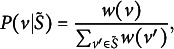



where a utility measure for answer choice *v* is represented as a positive real valued function 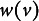 , which is directly proportional to the choice probability. In modeling a multiple-choice test item, utility measure can be considered a function of the latent ability of an examinee, 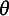 . Here, we take as the utility measure the probability of the option *v* being perceived as true by an examinee with ability level of 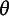 , i.e., 
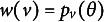
 . As we assume the IIA, 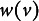 does not change after eliminating any other answer choice.

In sum, the IIA assumption implies that (1) the probability of making a choice can be expressed as a utility (or divide-by-total) model and thus gets re-scaled proportionally at every attempt. In other words, the probability of choosing an answer option is simply a scaling constant away from the utility measure of the option, and can thus be treated as interchangeable; and (2) eliminating an answer choice does not change the utilities of other alternatives, resulting in the unchanged relative choice probabilities. In the context of multiple attempts, we call the latter implication *attempt invariance*, which means that the utility measures will not change over attempts. This is a reasonable assumption as long as after every attempt, feedback is only given regarding their previous choice being correct or incorrect, without any additional information about the remaining answer choices. Later when we relax the attempt invariance assumption, we express the probability as 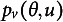 at attempt *u*, indicating that the probability depends on the number of attempts having been made.

To formulate the SIRT-MM model, we assume that 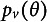 could be sufficiently modeled by a single latent variable 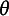 and item parameters. Let *T* be the correct answer choice and 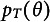 be the probability of considering the correct answer choice to be true. Let 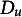 be the distractor with the *u*-th largest utility for each examinee and 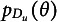 be the probability of endorsing the distractor at the *u*-th attempt, where each examinee would select in the order of 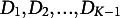 when they consistently make failed attempts. Note that we are not interested in specific choices of distractors for the ordering of 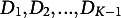 for each examinee, but we are only interested in modeling the expected *u*-th highest utility of a distractor at given 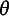 (i.e., 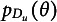 ), assuming that each examinee selects the answer choices with the highest utility subjectively judged by them.

Recall that, in SIRT, 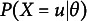 where 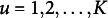 could be constructed by assuming 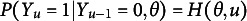 and 
(6)

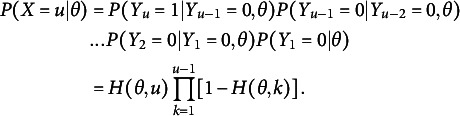



Then, based on the utility model presented in Eq. (5), the probability of submitting a correct answer on the first attempt is 
(7)

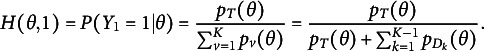



The conditional probability of submitting a correct answer at the second attempt is 
(8)

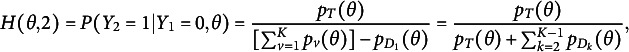



where a distractor 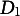 is initially mistakenly selected. This supports an intuition that 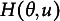 will be higher as *u* gets larger by eliminating distractors.

We begin by deriving the simplest form of an SIRT-MM model. This simple model assumes that all the distractors will are equally appealing to examinees, even after reattempts. In other words, we assume that all the distractors have the same probability of being selected given 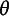 (i.e., homogeneity of distractors). Mathematically put, 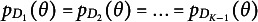 and we can simply denote 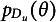 as 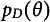 . One advantage of assuming both IIA and homogeneity of distractors is that it allows us not to assume a shape for 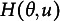 for 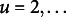 . In fact, 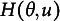 for 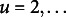 could be analytically derived from 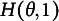 . Therefore, by assuming the shape of 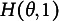 to be a 3PL logistic function, as the first attempt is technically the same as the 0/1 scoring, the whole model could be derived. Later in this article, we introduce parameterizations that allow us to relax both assumptions.

Now, for 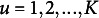 , 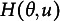 can be instead written as 
(9)

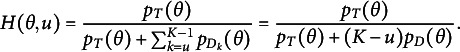



We can observe that the reciprocal of this probability, 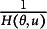 , decreases linearly by 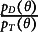 as *u* increases since: 
(10)

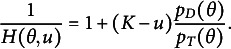



Finally, by assuming 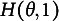 to be a 3PL logistic function with a fixed pseudo-guessing parameter of 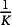 , which we denote as the “2.5 PL” model as it is between the 2PL and 3PL models (Bizot & Goldman, 1994), 
(11)

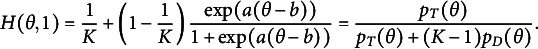



Thus, 
(12)

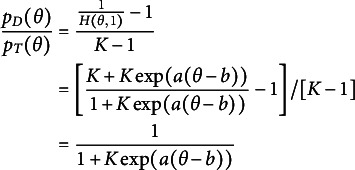



and 
(13)

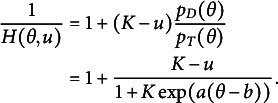



It is worth noting that we only model 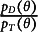 in the equations, instead of directly modeling 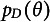 and 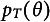 , respectively. Finally, the unconditional probability of choosing the correct choice for item *j* at the *u*-th attempt is derived as follows: 
(14)

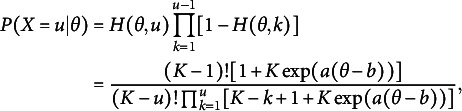



which is the simplest SIRT-MM model.

**Figure 1:**
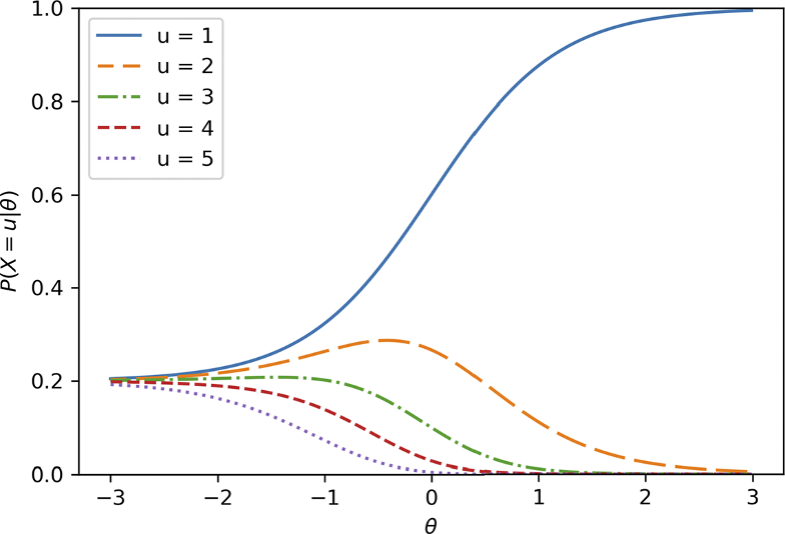
Item category response function: 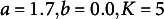 .

Figure 1 shows example item category response functions (i.e., 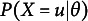 ) when 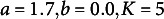 . As expected, we can observe that at any 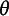 , 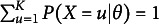 , and 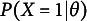 (or 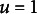 ) has the same shape as a 3PL logistic model. This figure also shows that conditioning on 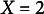 (or 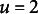 ), the middle range of 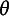 is the most likely when 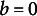 . This is intuitive as those who need exactly two attempts to get the right answer likely do not have very low or high 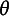 . Since we assume a fixed pseudo-guessing parameter of 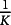 , 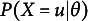 converges to 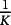 as 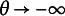 , suggesting complete ignorance will occur as 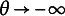 . This figure also shows that 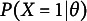 is the highest among all 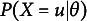 at any 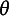 . To allow 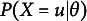 for 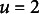 or above to be larger than 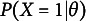 for some 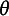 , we need to relax the homogeneity of distractors assumption and attempt invariance.

### 2.2 More general SIRT-MM models

Now, we turn to a more general case where distractors are not homogeneous, in particular, one distractor being the most attractive. Consider examinees with ability 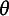 who try to evaluate all answer options of two test items. Let 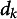 be the distractor *k* of an item, specified by its position within the item. Note that 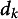 is different from 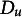 , which we introduced earlier to represent the distractor with the *u*-th largest utility for each examinee. Suppose on average examinees with ability 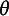 perceive the chance for the four options of item 1 being the correct choice as 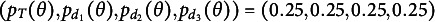 , and that of item 2 as 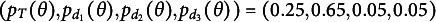 . Table 1 presents the probabilities of submitting a correct response at each attempt for the two items based on the utility model. Obviously, the probability of submitting a correct response at the first attempt is 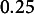 for both items. However, the chance of submitting a correct response at the second attempt is different. For the the first item, it is 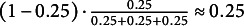 . For the second item it is 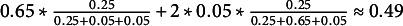 . Note that the first term of the second equation, which calculates the probability of choosing 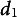 first and then the correct answer choice, is 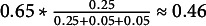 , meaning that when an examinee makes two attempts for the second item, they are likely tricked by the most attractive distractor, 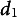 , and select 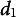 at the first attempt. These results also suggest that a multiple-attempt procedure penalizes complete ignorance more than partial (mis)information at the second attempt. For the first item, the person considers the correct answer choice to be equally likely, while for the second item, the person at least believes that the correct answer choice is more probable than 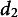 and 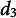 . In the second case, partial information helps avoid needing more than two attempts to answer the item correctly.

**Table 1:** Probabilities of submitting a correct response at each attempt for two hypothetical test items

Item with 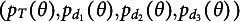	Condition	Attempt			
		1	2	3	4
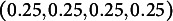	Complete ignorance	0.25	0.25	0.25	0.25
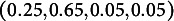	Partial (mis)information	0.25	0.49	0.21	0.05

We also consider another general case where the utility of any answer choice will change over reattempts. Suppose that the utility of answer choice *v* at attempt *u* is 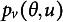 . The IIA assumption implies attempt invariance, which means that 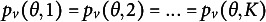 , allowing us to denote 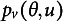 as 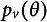 . However, this could be a strong assumption in a multiple-attempt procedure because the population changes after reattempts and specific characteristics of items might affect changes in 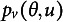 over reattempts. For example, a test item that requires certain factual knowledge (e.g., trivia questions) to answer might divide examinees into those who know the answer with confidence and those who do not know the answer at all. In such a case, conditioning on some 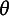 , the population could be divided into two groups by whether they know the requisite fact. For some value of 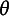 , the first group of examinees might believe 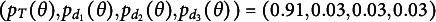 , while the second group of examinees might believe 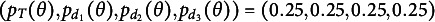 . If there is an equal number of examinees from each group in the population, on average, 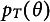 would be around 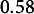 at the first attempt. However, after the first attempt, most likely only the latter group of examinees would proceed to the second attempt, leading to a much lower average 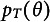 at the second attempt. A similar phenomenon was documented by Lyu et al. (2023)) in which they explain that certain item characteristics can have a larger effect after reattempts, resulting in higher difficulty estimates for multiple-attempt items.

To accommodate scenarios described above, we can formulate a more general SIRT-MM model which relaxes both the homogeneity of distractors and the attempt-invariance assumptions by introducing more parameters to vary the average utility of all the unattempted distractors relative to that of the correct answer choice across different attempts.

Recall that 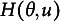 can be expressed as follows 
(15)

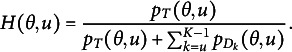



Thus, 
(16)

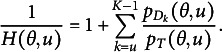



To model 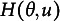 , we model the average of all the unattempted distractors at the *u*th attempt^2^: 
(17)

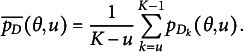



Then, 
(18)

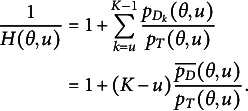



In modeling 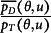 , as an extension of the simplest SIRT-MM model, which assumes 
(19)

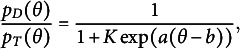



we propose to introduce the attempt-specific “difficulty-shift” parameter 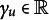 for 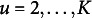 for a more general SIRT-MM model: 
(20)

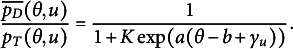



Therefore, 
(21)

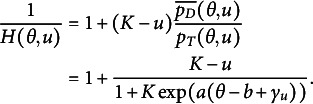



This leads to: 
(22)

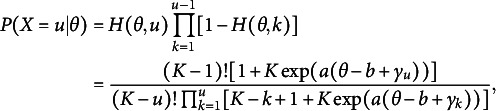



where 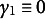 and 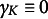 . This is the more general SIRT-MM model, of which the simplest SIRT-MM model (Eq. (14)) is a special case.

We define non-zero 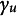 parameters only for 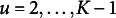 because: (a) 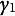 will lead to over-parameterization due to the existence of *b*, and (b) 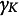 is not necessary since only one answer choice will be left after the 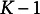 th attempt. Note that 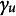 is item and attempt specific, but does not vary across examinees. We could further relax 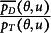 by allowing *a* to vary over each attempt at *u* (i.e., modeling 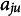 or 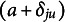 ). We discuss this extension in the Supplementary Material and discussion section.

#### Interpreting 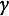 parameters

2.3

The simplest interpretation of 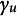 is that 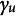 regulates the probability of making a successful attempt at the *u*th attempt. Specifically, when 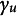 increases, 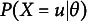 increases. Similarly, when 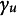 decreases, 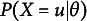 is decreased.

**Figure 2:**
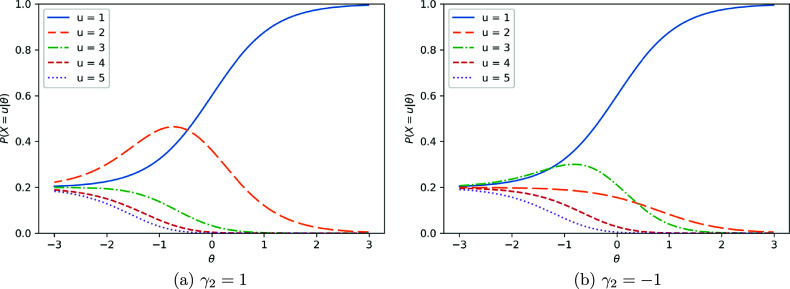
Item category response function: 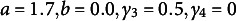 , and 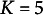 with different 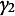 .

Figure 2 shows example item category response functions (ICRFs) when 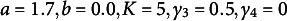 and two different 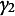 s. The left panel shows the ICRF with 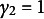 and the right panel shows the ICRF with 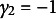 . All parameters except for the 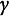 parameters are the same as those for Figure 1. Note that 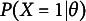 is unaffected by any 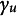 parameters, as non-zero 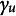 are only defined for 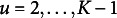 , which could not influence 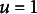 . The major difference between the two panels lies in 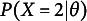 , which has a pronounced peak around 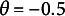 when 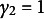 and is rather flat around 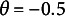 when 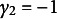 . As a result, 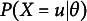 for 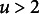 are also affected accordingly, which are smaller when 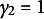 and larger when 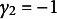 . This indicates that examinees with lower ability (e.g., 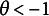 ) are more likely to require only two attempts to answer correctly when 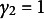 , whereas they are more likely to require three attempts when 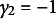 . Therefore, by adjusting 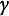 parameters, different types of item category response functions can be captured.

More specifically, 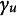 governs the change of probability ratio between the average of the unattempted distractors and the correct answer option and at the *u*th attempt (i.e., 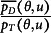 ) compared to the first attempt.

**Figure 3:**
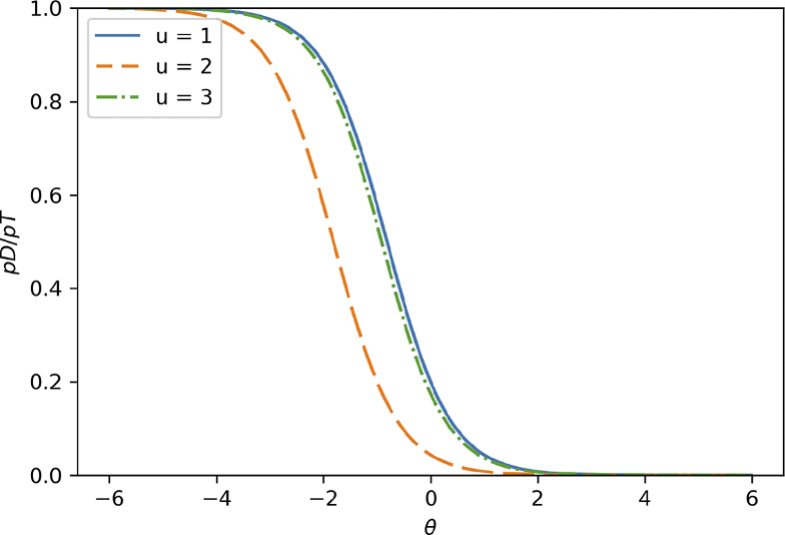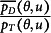 when 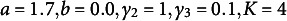 .

Figure 3 shows 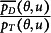 when 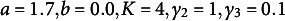 . We set 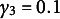 instead of 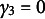 to prevent 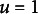 and 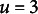 lines from overlapping. We can observe that 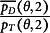 is shifted to the left by 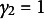 compared to 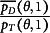 . Similarly, 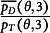 is shifted to the left by 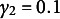 compared to 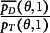

#### Possible factors that affect 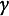 parameters

2.4

Increasing 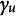 parameters over attempts can be caused by the heterogeneity of distractors. This means when 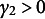 , 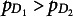 is expected. Furthermore, when 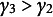 , 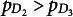 is expected. For example, when 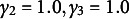 , and 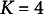 for an item, there is at least one attractive distractor that will make examinees more likely to make two attempts. Numerically, a positive 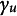 reduces 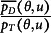 at the *u*th attempt. Logically, increasing 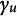 indicates knowledge gain through correcting partial misinformation because after a failed attempt, a distractor with high utility will be eliminated, leading to 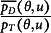 to be smaller at later attempts and the correct answer option even more appealing. Therefore, increasing 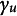 could signify the heterogeneity of distractors. Increasing 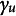 might also be caused by informative feedback such as hints after a wrong response.

On the other hand, as we described earlier, decreasing 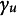 parameters can be caused by the population changes after reattempts and specific characteristics of items. In the example of an item requiring factual knowledge, 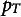 could decrease because the examinees who fails the first attempt would likely fail the second attempt as well, and they represent the majority of those who need the second attempt. Thus, 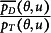 would increase from the first to second attempt, which would be captured by a negative 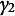 .

In addition, negative 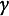 parameters could result from having a large number of examinees who are being inattentive and fail to eliminate already selected distractors in reattempts. In this article, we assume that examinees are attentive. However, if the system allows examinees to select the same wrong answer option repeatedly, negative 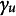 could result.

#### 2.5 Setting the maximum number of attempts

One advantage of using an SIRT model is that we can limit the maximum number of attempts in a test item, as it is not influenced by attempt-specific parameters such as 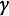 parameters from later attempts (Tutz, 1990). This is especially useful when a sample size is not large enough to reliably estimate attempt-specific parameters for later attempts. In addition, thanks to the future-agnostic property of SIRT models, we can also reuse the same item parameters and collapse certain categories when only a smaller number of attempts is allowed.

**Figure 4:**
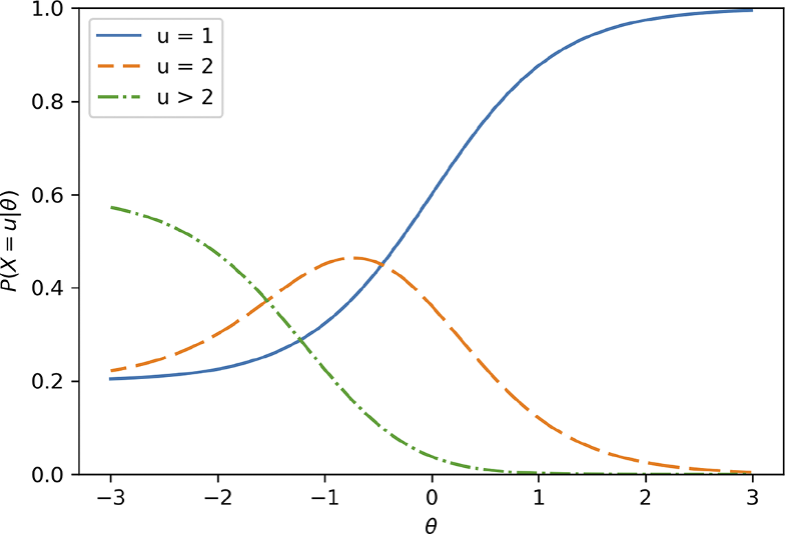
Item category response function when the maximum number of attempts is 3: 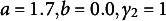 , and 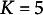 .

For example, Figure 4 shows the item category response functions used in Figure 2a when we set the maximum number of attempts to three. Simply, these are the item category response functions shown in Figure 2a, but 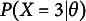 , 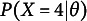 , and 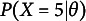 are collapsed into one category. In this example, only 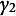 is relevant, and no matter what true value of 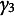 or 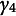 would be, the SIRT-MM model yields exactly the same model when the maximum number of attempts is three.

#### 2.6 Summary of different parameterizations of SIRT-MM models

Bergner et al. (2019)) summarized existing SIRT models in a table. Similarly, we summarize in Table 2 a family of SIRT-MM models with different constraints. We denote the subject *j* for individual items and *u* for the number of attempts. The number of parameters depends on constraints imposed or lifted. The basic SIRT-MM model introduced first in this article is actually a constrained version of the more general SIRT-MM models when 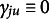 . We recommend that a model should be selected based on the sample size and model fit statistics such as likelihood ratio tests, Akaike information criterion (AIC; Akaike, 1973) and Bayesian information criterion (BIC; Schwarz, 1978). Later we evaluate the accuracy of model selection using AIC and BIC in the simulation study section.

**Table 2:** Family of SIRT-MM models

Constraint	No. of parameters	Description
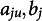 , and 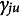 are unconstrained	2M(K-1)	The SIRT-MM model with the highest degrees of freedom
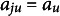	(M + 1)(K-1)	
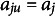	MK	The second SIRT-MM model we formulated (Eq. (22))
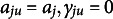 for all 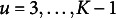	3M	A reduced version of the second SIRT-MM model
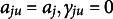 for all 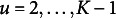	2M	The simplest SIRT-MM model we formulated (Eq. (14)) 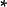
⋮		
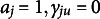	M	The simplest SIRT-MM model with fixed 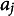 parameters 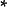

*Note*: The second column shows the number of item parameters where *M* is the number of items and *K* is the number of answer choices. The models with * at the end of description have the homogeneity of distractors and attempt-invariance assumptions.

### 2.7 Item parameter estimation of SIRT-MM

In this article, we implement marginal maximum likelihood estimation (MMLE) for estimating the item parameters for SIRT-MM models (Bock & Aitkin, 1981). Once the item parameters are estimated, we will estimate 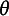 after treating estimated item parameters as fixed.

In MMLE for item parameters, the likelihood function, 
(23)

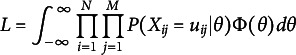



is maximized, where 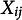 is a random variable representing the number of attempts examinee *i* needed to submit a correct answer on item *j*, 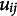 is the actual number of attempts taken by examinee *i* to answer item *j* correctly, *N* is the number of examinees, *M* is the number of items, and 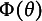 is a probability density function for the population. Typically, the standard normal distribution is used for 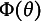 .

To maximize the likelihood function, we use the log-likelihood, denoted as 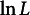 , instead. Consequently, we require the gradient and Hessian of 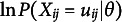 with respect to a parameter of interest to apply Newton’s method for maximizing the likelihood function. However, computing the value of the log-likelihood function is not straightforward because the equation contains an integral. In practice, an EM algorithm that uses Gauss–Hermite quadratures is used to compute the marginal likelihood. One should refer to the works by Bock and Aitkin (1981); Muraki (1992) for the details of implementing an EM algorithm for parameter estimation. We will suppress the subscript *i* next for simplicity.

The generic solutions of the gradient and Hessian of 
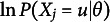
 are rather straightforward. Suppose 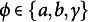 and 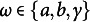 are the parameters of interest: 
(24)

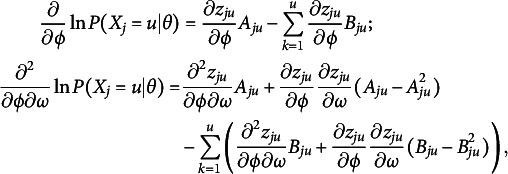



where 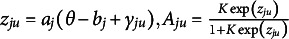 , and 
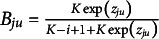
 . Especially, 
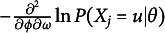
 is called the observed information function and 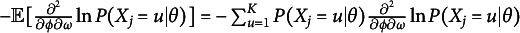 is called the expected or Fisher information function of an item. In addition, the Fisher information function of an item is often simply referred to as an item information function. When we estimate a simple model with fewer parameters by setting 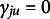 for any *u*, we only need to set these values to zero in 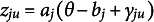 and use the same equations, Eq. (24).

### 2.8 Person parameter estimation

There exist three popular approaches for estimating person parameters: maximum likelihood estimation (MLE), maximum A posteriori (MAP), and expected A posteriori (EAP) (De Ayala, 2009). MLE maximizes the log-likelihood of a response pattern by Newton’s method, MAP uses the mode of the posterior distribution of an 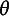 estimate (typically the standard normal distribution is used for prior), and EAP uses the mean of the posterior distribution of an 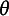 estimate (De Ayala, 2009). In our model, MLE could be obtained by maximizing 
(25)

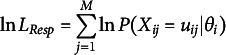



with respect to 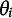 where 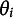 is the latent ability of examinee *i*, which could be done by Newton’s method using Eq. (25). One issue in using MLE is that it cannot provide a 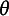 estimate when a response pattern is all 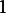 or *K*. Also, it is known that the mean squared error of 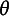 estimates by EAP is smaller than that obtained by using MLE although its estimation bias is increased (De Ayala, 2009; Lord, 1986). Thus, we use EAP in our simulation study.

### 2.9 Fisher information and standard errors

Under regularity conditions, the Fisher information of item parameter 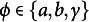 is 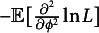 and that of 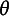 is 
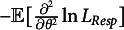
. Thus, we can calculate the standard errors of estimates in 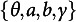 , which is inversely related to the square root of the corresponding Fisher information. Thus, the standard error of item parameter 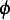 is 
(26)

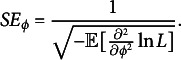



Similarly, the standard error of measurement (SEM), which is the standard error of 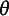 is 
(27)

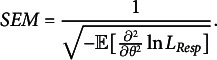



However, the SEM as defined in the above formula is based on MLE. In this study, since we use EAP, we decide to capture the variation in the person parameter estimates using the empirical SE instead.

### 2.10 Item information

**Figure 5:**
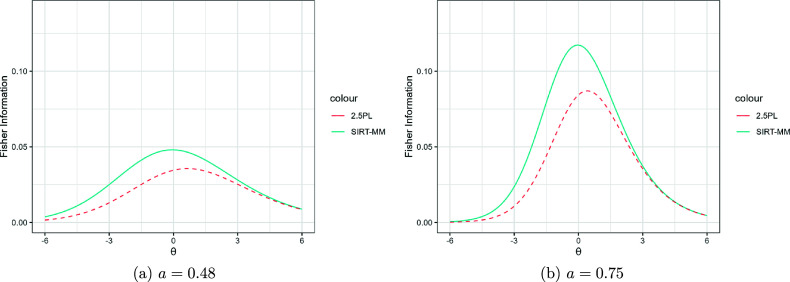
Fisher information of SIRT-MM models with 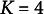 , 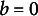 , and 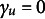 for 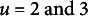 , and different *a*; and the corresponding 2.5PL models with 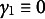 .

Item information is Fisher information computed with respect to 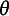 for any single item, which is a measure of how much an item contributes to reducing the uncertainty about 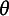 estimates (De Ayala, 2009). We can compare item information computed by using SIRT-MM models (which captures information from multiple attempts) against its corresponding 2.5PL model (i.e., the 3PL model with a fixed guessing parameter of 1/K) to demonstrate how much SIRT-MM models potentially improve the accuracy of 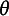 estimates. For example,

Figure 5 shows the item information of SIRT-MM models with 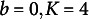 , and 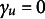 for 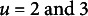 , two levels of *a* parameters ( 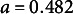 in the left panel and and 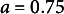 in the right panel), and its corresponding 2.5PL model. As with the 2.5PL model, SIRT-MM models provide more Fisher information as the *a* parameter increases. It is noteworthy that for lower 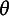 , SIRT-MM models can yield more information than their 2.5PL counterparts. This is because though reattempts we can gain more information about examinees who fail the first attempt, which is more likely to happen for examinees with lower 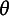 . Conversely, for higher 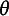 , both models have similar information because examinees with higher 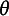 typically only need one attempt to reach the correct answer.

**Figure 6:**
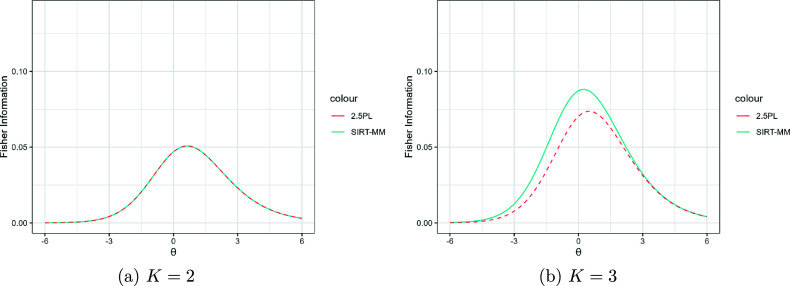
Fisher information of SIRT-MM models with 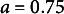 , 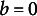 , 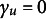 for 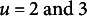 , and different *K*; and the corresponding 2.5PL models with 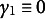 .

Figure 6 shows the item information of SIRT-MM models with 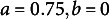 , and 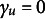 for 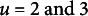 , two levels of *K* parameters ( 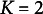 in the left panel and and 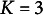 in the right panel), and its corresponding 2.5PL model. The left panel demonstrates that the 2.5PL models can be considered a special case of SIRT-MM models when 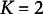 . Also, the comparison between the two panels shows that the item information increases for both 2.5PL and SIRT-MM models when *K* increases because the chance of guessing is reduced.

**Figure 7:**
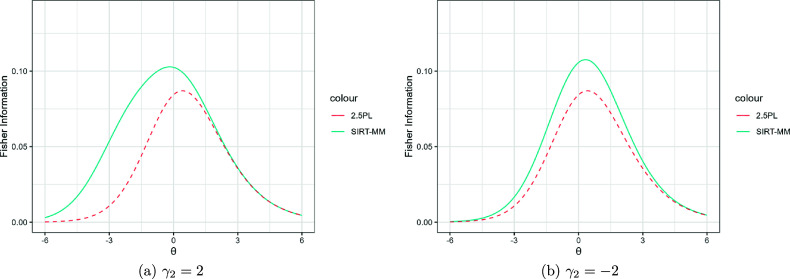
Fisher information of SIRT-MM models with 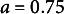 , 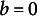 , 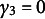 , 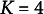 , and different 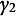 ; and the corresponding 2.5PL models with 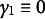 .

Figure 7 shows the item information of SIRT-MM models with 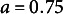 , 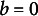 , and 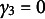 for 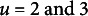 , 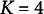 , two levels of 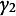 ( 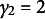 on the left, and 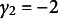 on the right), and the corresponding 2.5PL models. Changing the 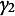 will affect the amount of item information in lower 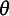 . When 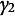 is positive, we can gain more item information in lower 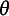 than when it is negative because in the latter case examinees with lower 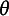 would not be able to differentiate among distractors and behave similarly to random guessing after the first attempt, as in the example of factual knowledge item.

## 3 Simulation studies

### 3.1 Simulation design

We conducted three simulation studies on: model selection, item parameter recovery, and person parameter recovery, respectively. A high-level description of the simulation design shared by these simulation studies is provided here. First, we generated response matrices from the SIRT-MM models. Second, item parameters were estimated by MMLE using an EM algorithm (Bock & Aitkin, 1981) implemented in R and C++. We provide the R package on GitHub https://github.com/luyikei/sirtmm to fit the SIRT-MM models. A standard normal prior was used for 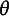 in MMLE. Third, with the estimated item parameters considered fixed, person parameters were estimated by EAP. Here, a standard normal prior was used for 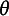 again. Generally, our simulation design followed Reise and Yu (1990).

The first simulation evaluated model selection using AIC and BIC to identify the best model, among SIRT-MM models with all combinations of freely estimated 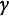 parameters to fit multiple attempt data. In addition, we also compared SIRT-MM models in terms of model fit against Graded response model (GRM; Samejima, 1969), since previous research showed that GRM could also be used for AUC (Attali, 2011), as well as Nominal response model (NRM; Bock, 1972). The simulation conditions are specified as 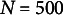 and 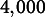 , 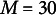 , 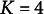 , 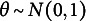 , 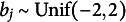 , 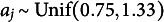 , and 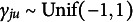 . We analyzed the impact of varying sample sizes, 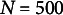 and 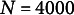 , to assess how differences in sample size influence model selection performance. For each replication, we simulated responses from all possible SIRT-MM models and fit all candidate models (SIRT-MM, GRM, and NRM models) to each response matrix. Over 100 replications, we were able to obtain model selection with AIC and BIC.

Then, we conducted simulation studies to evaluate item and person parameter recovery of SIRT-MM models. As item and person parameter estimations take place at different stages, we evaluated them separately. The second simulation study investigates item parameter recovery of SIRT-MM models under different sample sizes and test length conditions. The number of answer choices is 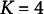 . We evaluated all the combinations of (1) the sample size: 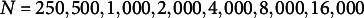 ; (2) the number of items: 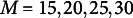 ; and (3) the number of 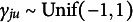 . We also evaluated more simulation conditions varying other factors; however, they are not fully crossed with conditions (1)–(3), as it would result in an unrealistic number of simulation conditions. Specifically, we evaluated conditions varying (4) 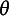 distribution: normal ( 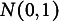 ), uniform ( 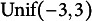 ), and skewed normal distribution 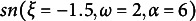 using sn package (Azzalini, 2022); (5) item discrimination parameter, 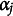 : sampled from low ( 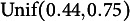 ), middle ( 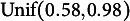 ), high ( 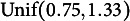 ), and all ( 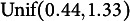 ) ranges; (6) item difficulty parameter: sampled from all ( 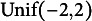 ) and high ( 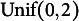 ) ranges; and (7) setting the maximum number of attempts of 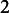 vs. 4. The simulation conditions in (4)–(7) are evaluated with different sample sizes but share the same baseline condition, which is specified as follows: 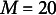 , 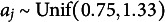 , 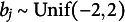 , for 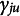 parameters, only 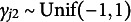 is specified as a freely estimated parameter, and the 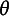 distribution is the standard normal distribution ( 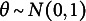 ). The skewed distribution for 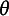 is positively skewed in order to evaluate the performance of person parameter recovery for a low-ability population, for which the SIRT-MM models are good candidates. For the same reason, the item difficulty parameter has a condition where only relatively difficult items exist. The convergence rate of item parameter estimation was reported for each simulation condition. Standard errors (SE) for the item parameters, bias, and root mean square error (RMSE) were used as primary indices to examine the quality of parameter estimates, which were obtained for the converged conditions. Out of 100 replications, we calculated the averages of metrics from all converged replications across all conditions.

The third simulation study evaluated person parameter recovery following the same baseline condition as the first simulation: 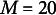 , 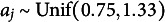 , 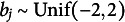 . For 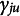 parameters, only 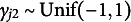 is specified as a freely estimated parameter, and the 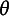 distribution is the standard normal distribution ( 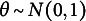 ). 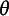 was estimated by EAP treating item estimates as fixed. To evaluate person parameter recovery, we used the 2.5PL model as a baseline for comparison since multiple-attempt responses could be converted to 0/1 scoring if we only take first-attempt data. Note that model fit and selection are evaluated in a different simulation study and the purpose of this comparison is to show how much improvement in person parameter recovery could be gained by just allowing multiple attempts using the same test items. We used mirt package for estimating the 2.5PL model (Chalmers, 2012). In addition to bias and RMSE, the Pearson correlation coefficient was also used to assess the recovery accuracy for 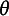 . When we evaluate correlation, we also included the results of a popular scoring scheme in CTT which grants full credit for the successful first attempt to an item, half credit for the successful second attempt, one-tenth credit for the successful third attempt, and zero credit otherwise (Slepkov & Godfrey, 2019). Results were presented by taking the averages of metrics calculated from 100 replications for all conditions.

### 3.2 Results

### 3.3 Model selection

**Figure 8:**
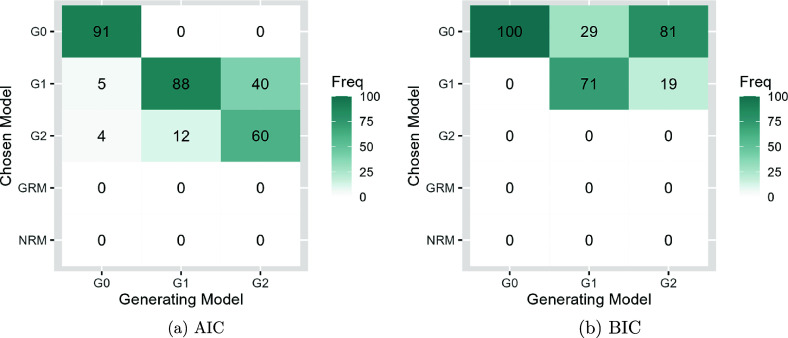
Model selection performance of AIC and BIC for SIRT-MM models when data are generated from SIRT-MM models with 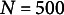 , 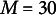 , 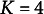 , 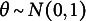 , 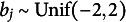 , 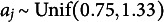 , and 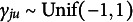 . The freely estimated 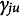 are denoted as *Ga* where *a* is the number of 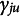 parameters for all *u*.

**Figure 9:**
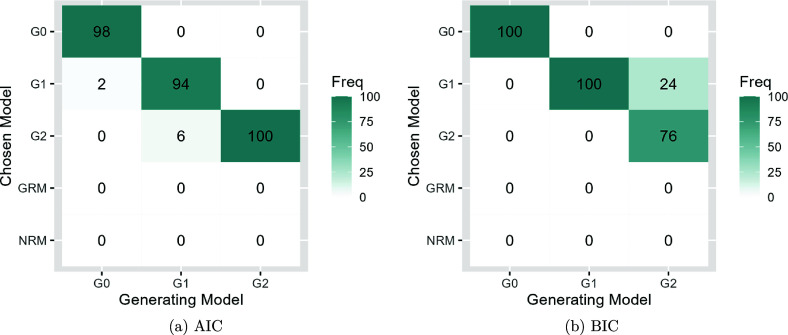
Model selection performance of AIC and BIC for SIRT-MM models when data are generated from SIRT-MM models with 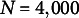 , 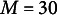 , 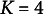 , 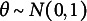 , 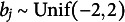 , 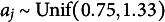 , and 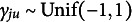 . The freely estimated 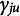 are denoted as *Ga* where *a* is the number of 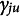 parameters for all *u*.

Figures 8 and 9 present the model selection performance of AIC and BIC for SIRT-MM models with 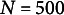 and 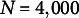 . First, both AIC and BIC successfully select an SIRT-MM model over GRM or NRM when responses are simulated from an SIRT-MM model. Second, for selecting the correct SIRT-MM model from all the variants of SIRT-MM models, AIC selects the correct model the majority of times regardless of *N*, and both AIC and BIC perform well with larger *N*. Specifically, when 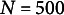 , AIC could identify the correct model about 90% of the time and BIC could identify the correct model about 86% of the time from models with or without 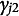 . However, for the data generating model incorporating both 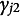 and 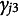 , a sample size of N = 500 results in AIC correctly identifying the model 60% of the time, while BIC never identifies the correct model. When 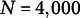 , AIC could identify the correct model about 97% of the time and BIC could identify the correct model about 92% of the time from all the models. Between the two, AIC seems to outperform BIC, as BIC could under-specify the model, though in a small number of cases AIC could over-specify the model. Specifically, with 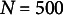 , in 21 out of a total of 300 generated cases across the conditions AIC over-specified the model while BIC under-specifies the model with 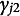 in 29 out of 100 cases and consistently under-specifies the model with both 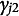 and 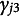 (i.e., 100 out of 100 cases). In contrast, AIC under-specifies the model containing both 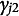 and 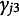 in 40 out of 100 cases. When 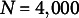 , AIC identifies 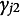 parameters when no 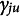 was included in the generating model in 2 out of 100 cases, and AIC identifies 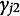 and 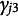 when the generating model only had 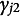 parameter in 6 out of 100 cases. On the other hand, it is worth noting that there are 24 out of all the generated cases (300) across the conditions where BIC identifies only 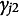 parameters while the data generating model includes both 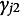 and 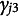 . In our simulation results under both sample size conditions, AIC is more accurate in selecting the true model and BIC in some cases picks an over-simplified model.

### 3.4 Item parameter recovery

Tables 3–5 present the item parameter recovery statistics varying sample size, number of items, and number of effective 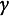 parameters when 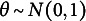 , 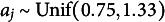 , 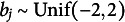 . The results show that, as *N* gets larger, the SE and RMSE for the item parameter estimates decrease and the bias quickly converges to zero in all the conditions, suggesting that our estimation method could yield satisfactory item parameter recovery for all conditions given a large enough *N*. Although *M* has a smaller effect on item recovery statistics compared to *N*, generally larger *M* also leads to better item parameter estimates.

**Table 3:** Item recovery statistics for items without 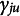

		SE		BIAS		RMSE		CONV
*M*	*N*	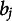	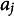	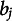	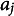	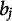	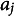	
15	250	0.30	0.23	0.02	0.02	0.40	0.32	1.00
	500	0.18	0.16	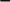 0.00	0.01	0.23	0.21	1.00
	1,000	0.12	0.11	0.01	0.01	0.16	0.15	1.00
	2,000	0.09	0.08	0.01	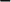 0.00	0.12	0.11	1.00
	4,000	0.06	0.06	0.00	0.00	0.08	0.07	1.00
	8,000	0.04	0.04	0.01	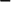 0.00	0.06	0.05	1.00
	16,000	0.03	0.03	0.01	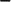 0.00	0.04	0.04	1.00
20	250	0.30	0.23	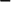 0.00	0.03	0.40	0.31	0.99
	500	0.19	0.16	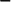 0.01	0.00	0.25	0.20	1.00
	1,000	0.12	0.11	0.01	0.00	0.16	0.15	1.00
	2,000	0.09	0.08	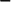 0.00	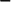 0.00	0.11	0.10	1.00
	4,000	0.06	0.06	0.00	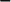 0.00	0.08	0.07	1.00
	8,000	0.04	0.04	0.00	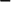 0.00	0.05	0.05	1.00
	16,000	0.03	0.03	0.01	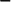 0.01	0.04	0.04	1.00
25	250	0.28	0.23	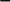 0.00	0.02	0.36	0.29	1.00
	500	0.18	0.16	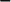 0.01	0.01	0.23	0.20	1.00
	1,000	0.12	0.11	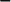 0.00	0.00	0.15	0.14	1.00
	2,000	0.09	0.08	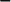 0.00	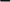 0.00	0.10	0.09	1.00
	4,000	0.06	0.06	0.01	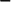 0.00	0.07	0.07	1.00
	8,000	0.04	0.04	0.00	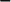 0.01	0.05	0.05	1.00
	16,000	0.03	0.03	0.00	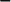 0.01	0.04	0.03	1.00
30	250	0.28	0.23	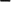 0.00	0.02	0.36	0.29	1.00
	500	0.18	0.16	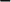 0.01	0.01	0.23	0.20	1.00
	1,000	0.12	0.11	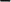 0.00	0.00	0.16	0.14	1.00
	2,000	0.09	0.08	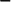 0.00	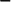 0.00	0.10	0.10	1.00
	4,000	0.06	0.06	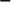 0.00	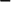 0.01	0.07	0.07	1.00
	8,000	0.04	0.04	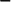 0.00	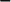 0.01	0.05	0.05	1.00
	16,000	0.03	0.03	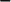 0.00	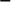 0.01	0.04	0.03	1.00

*Note*: “CONV” stands for convergence rate for a simulation condition.

**Table 4:** Item recovery statistics for items with 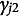

		SE			BIAS			RMSE			CONV
*M*	*N*	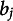	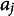	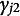	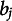	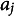	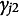	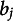	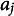	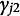	
15	250	0.37	0.23	0.55	0.03	0.03	0.03	0.52	0.33	1.00	0.98
	500	0.20	0.16	0.35	0.01	0.03	0.01	0.26	0.24	0.49	1.00
	1,000	0.13	0.11	0.24	0.01	0.01	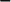 0.01	0.17	0.15	0.27	1.00
	2,000	0.09	0.08	0.17	0.01	0.00	0.00	0.12	0.11	0.18	1.00
	4,000	0.06	0.06	0.11	0.01	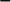 0.00	0.00	0.08	0.08	0.12	1.00
	8,000	0.05	0.04	0.08	0.01	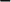 0.00	0.00	0.06	0.05	0.09	1.00
	16,000	0.03	0.03	0.06	0.01	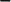 0.00	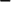 0.00	0.04	0.04	0.06	1.00
20	250	0.30	0.24	0.51	0.01	0.05	0.06	0.39	0.32	0.78	0.95
	500	0.20	0.16	0.34	0.01	0.02	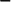 0.00	0.25	0.22	0.36	1.00
	1,000	0.13	0.11	0.24	0.00	0.00	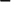 0.01	0.17	0.15	0.27	1.00
	2,000	0.09	0.08	0.17	0.01	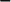 0.00	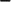 0.00	0.12	0.10	0.18	1.00
	4,000	0.06	0.06	0.12	0.01	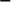 0.00	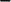 0.00	0.08	0.07	0.13	1.00
	8,000	0.05	0.04	0.08	0.00	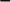 0.00	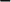 0.00	0.06	0.05	0.09	1.00
	16,000	0.03	0.03	0.06	0.00	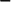 0.00	0.00	0.04	0.04	0.06	1.00
25	250	0.30	0.24	0.52	0.01	0.03	0.04	0.37	0.31	0.77	0.97
	500	0.20	0.16	0.35	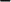 0.00	0.01	0.01	0.24	0.21	0.43	1.00
	1,000	0.13	0.11	0.24	0.00	0.00	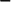 0.00	0.16	0.14	0.25	1.00
	2,000	0.09	0.08	0.17	0.01	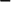 0.00	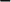 0.00	0.11	0.10	0.18	1.00
	4,000	0.06	0.06	0.12	0.00	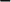 0.00	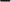 0.00	0.08	0.07	0.12	1.00
	8,000	0.05	0.04	0.08	0.00	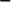 0.01	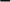 0.00	0.06	0.05	0.09	1.00
	16,000	0.03	0.03	0.06	0.00	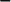 0.01	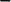 0.00	0.04	0.04	0.06	1.00
30	250	0.30	0.23	0.58	0.00	0.02	0.07	0.38	0.30	1.11	0.97
	500	0.20	0.16	0.35	0.00	0.01	0.00	0.24	0.20	0.38	1.00
	1,000	0.13	0.11	0.24	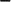 0.00	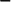 0.00	0.01	0.16	0.14	0.25	1.00
	2,000	0.09	0.08	0.17	0.01	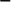 0.00	0.00	0.11	0.10	0.18	1.00
	4,000	0.07	0.06	0.12	0.00	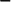 0.01	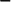 0.00	0.08	0.07	0.12	1.00
	8,000	0.05	0.04	0.08	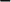 0.00	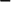 0.01	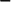 0.00	0.05	0.05	0.09	1.00
	16,000	0.03	0.03	0.06	0.00	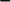 0.01	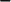 0.00	0.04	0.03	0.06	1.00

*Note*: “CONV” stands for convergence rate for a simulation condition.

**Table 5:** Item recovery statistics for an item with 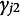 and 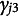

		SE				BIAS				RMSE				CONV
*M*	*N*	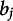	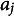	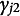	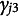	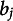	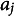	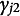	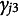	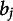	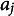	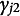	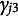	
15	250	0.30	0.24	0.51	1.03	0.04	0.07	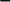 0.03	1.97	0.39	0.34	0.92	7.30	0.67
	500	0.19	0.17	0.34	0.67	0.02	0.03	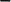 0.00	0.94	0.24	0.24	0.49	4.32	0.89
	1,000	0.14	0.12	0.24	0.49	0.01	0.01	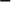 0.00	0.43	0.18	0.16	0.27	2.02	0.97
	2,000	0.10	0.08	0.17	0.34	0.01	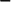 0.00	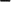 0.00	0.07	0.13	0.12	0.19	0.62	1.00
	4,000	0.07	0.06	0.12	0.25	0.01	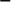 0.01	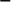 0.01	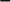 0.00	0.09	0.08	0.13	0.29	1.00
	8,000	0.05	0.04	0.08	0.17	0.01	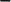 0.00	0.00	0.00	0.06	0.06	0.09	0.19	1.00
	16,000	0.03	0.03	0.06	0.12	0.01	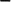 0.00	0.00	0.01	0.04	0.04	0.06	0.14	1.00
20	250	0.32	0.24	0.53	1.05	0.03	0.06	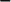 0.01	2.05	0.44	0.36	1.01	8.57	0.53
	500	0.20	0.17	0.35	0.70	0.02	0.01	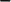 0.02	0.91	0.26	0.23	0.38	5.01	0.89
	1,000	0.14	0.12	0.24	0.49	0.01	0.01	0.00	0.48	0.17	0.16	0.26	2.57	0.99
	2,000	0.10	0.08	0.17	0.35	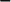 0.00	0.00	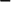 0.01	0.09	0.12	0.11	0.18	0.78	0.99
	4,000	0.07	0.06	0.12	0.24	0.01	0.00	0.00	0.01	0.08	0.08	0.13	0.33	1.00
	8,000	0.05	0.04	0.08	0.17	0.00	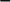 0.00	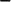 0.00	0.00	0.06	0.05	0.09	0.18	1.00
	16,000	0.03	0.03	0.06	0.12	0.00	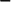 0.00	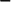 0.00	0.00	0.04	0.04	0.06	0.13	1.00
25	250	0.31	0.24	0.53	0.97	0.03	0.05	0.08	2.39	0.39	0.33	1.16	8.96	0.51
	500	0.20	0.17	0.35	0.69	0.01	0.03	0.03	1.18	0.25	0.22	0.47	5.34	0.90
	1,000	0.14	0.12	0.24	0.49	0.00	0.00	0.01	0.39	0.16	0.15	0.26	2.12	0.97
	2,000	0.10	0.08	0.17	0.34	0.01	0.00	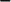 0.00	0.01	0.12	0.10	0.18	0.39	0.99
	4,000	0.07	0.06	0.12	0.25	0.00	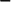 0.00	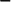 0.00	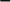 0.00	0.08	0.07	0.12	0.28	0.99
	8,000	0.05	0.04	0.08	0.17	0.00	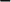 0.01	0.00	0.00	0.06	0.05	0.09	0.19	1.00
	16,000	0.03	0.03	0.06	0.12	0.00	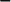 0.01	0.00	0.00	0.04	0.04	0.06	0.13	1.00
30	250	0.29	0.24	0.51	0.97	0.00	0.05	0.05	2.10	0.35	0.31	0.86	8.87	0.44
	500	0.20	0.16	0.36	0.69	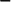 0.00	0.01	0.01	0.97	0.25	0.21	0.43	4.93	0.89
	1,000	0.14	0.12	0.24	0.51	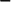 0.00	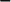 0.00	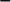 0.00	0.31	0.16	0.14	0.26	2.14	0.98
	2,000	0.10	0.08	0.17	0.35	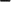 0.00	0.00	0.00	0.10	0.11	0.10	0.18	0.78	0.99
	4,000	0.07	0.06	0.12	0.24	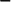 0.00	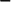 0.01	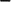 0.00	0.01	0.08	0.07	0.13	0.33	1.00
	8,000	0.05	0.04	0.08	0.17	0.00	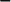 0.01	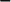 0.00	0.01	0.06	0.05	0.09	0.20	1.00
	16,000	0.03	0.03	0.06	0.12	0.00	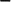 0.01	0.00	0.00	0.04	0.04	0.06	0.13	1.00

*Note*: “CONV” stands for convergence rate for a simulation condition.

Different SIRT-MM models tend to vary in their sample size requirements, and thus investigated separately. Table 3 presents item parameter recovery results when all 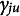 parameters are constrained to be zero. This model has the fewest number of item parameters among all variants, as only 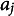 and 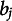 are estimated. Please note that this is not the same as a 2.5PL model, because we simulated a maximum of four attempts. Item parameter estimation converged in all conditions, except for one single case when 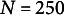 and 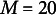 . The RMSE for the item parameter estimates are smaller than 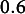 in all *N* and *M* conditions, smaller than 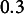 with 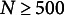 , and could be smaller than 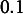 with 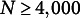 . Overall, these results show that the item parameters from the simplest SIRT-MM model can be recovered very well when the model fits the data with a reasonable sample size (e.g., 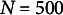 or more).

Table 4 presents item recovery statistics when only 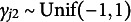 is specified as a freely estimated parameter. Item parameter estimation generally converged in all conditions although when 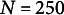 , there is a 2%–5% chance of non-convergence. The RMSE for the item parameter estimates are smaller than 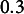 when 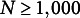 . However, if we can compromise the accuracy of 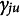 a little bit, 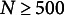 is also acceptable since the RMSE for 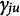 will not be larger than 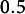 when 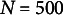 . Table 5 presents item recovery statistics when 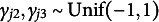 are specified as freely estimated parameters. We do not recommend 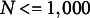 for estimating both 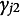 and 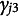 because the RMSE for 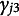 are generally very high and the convergence rates could be low. On the other hand, the RMSE for all the item parameters are smaller than 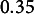 when 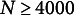 .

In Supplementary Material, we include additional tables, Supplementary Tables S1–S4, which present the item parameter recovery statistics varying 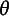 distributions, item discrimination parameters, item difficulty parameters and the maximum number of attempts respectively. Especially, Supplementary Table S1 shows that an SIRT-MM models works better with a positively skewed 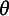 distribution than the 2.5PL model, and Supplementary Table S3 shows that having a relatively difficult test (by keeping 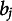 parameters to a high range) does not seem to affect item parameter estimation much for SIRT-MM models. This is because SIRT-MM models can glean more item information in the lower 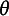 range from multiple attempts. Please refer to the Supplementary Material for further elaboration.

In sum, although all the factors more or less affect the accuracy of item parameter estimates, having a reasonably large sample size enables quality item parameter estimates. For a simple SIRT-MM model, we recommend 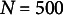 or more. For more complex SIRT-MM models, 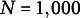 or 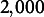 or more might be needed.

### 3.5 Person parameter recovery

**Figure 10:**
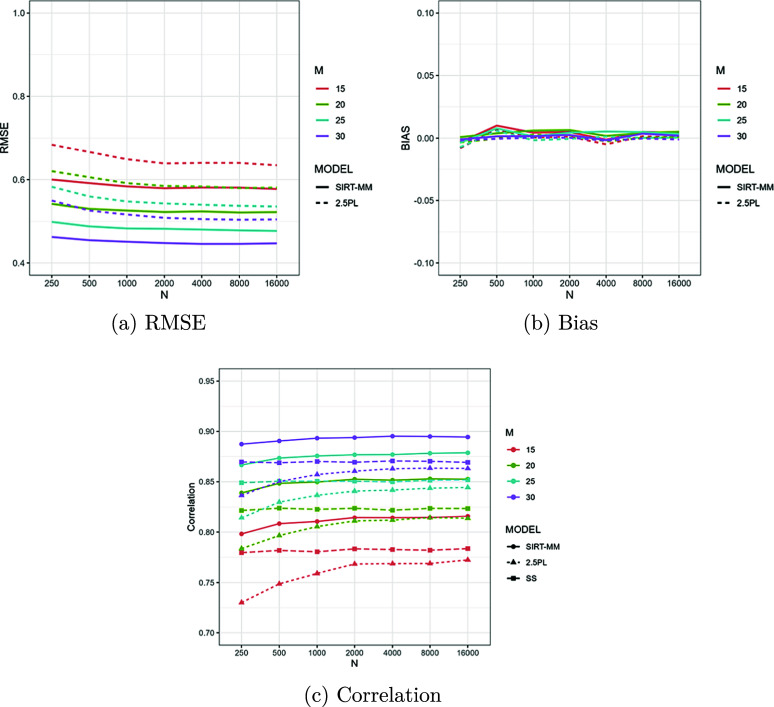
Person parameter statistics when 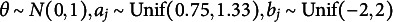 , and 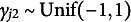 . *M* is the number of items administered. The scoring scheme used in classical test theory is denoted as SS in the correlation plot.

Figure 10 shows the person recovery statistics varying the number of items, *M*, when 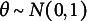 , 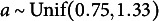 , 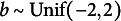 , and 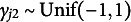 . The left panel shows RMSE, the middle panel shows bias and the right panel shows correlations between the true and estimated 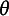 . The RMSE for 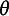 estimated by the SIRT-MM model with freely estimated 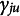 parameters is consistently smaller than those estimated by the 2.5PL model. The RMSE of 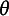 estimates is quite low even with 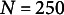 , indicating that the person parameter estimation can be robust even at very small sample sizes. The bias for 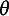 estimated both by the SIRT-MM model and the 2.5 PL model are consistently close to zero. The correlations between true 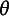 and 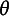 estimated by the SIRT-MM model are consistently the highest among the three scoring mechanisms. Typically the CTT scoring scheme outperforms the 2.5PL model in terms of correlation because it still can recover some partial information from multiple attempts.

**Figure 11:**
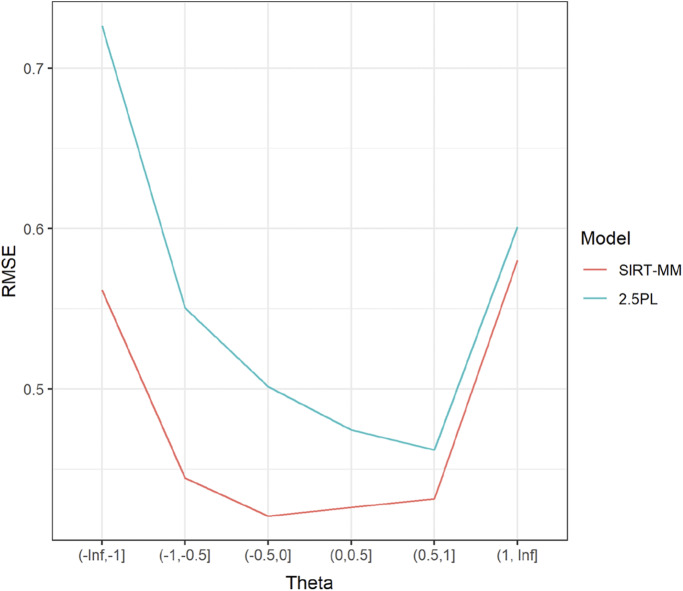
RMSE for 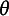 estimates conditioning on 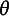 when 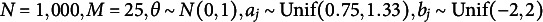 .

Figure 11 shows the conditioned RMSE for 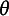 estimates. The SIRT-MM model leads to lower RMSE, especially at the lower range of 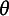 . Thus, the SIRT-MM model could be used for improving person parameter estimates, especially at the low end of the 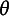 .

In Supplementary Material, we include additional figures, Supplementary Figures S3–S6, which present the person parameter recovery statistics varying 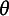 distributions, item discrimination parameters, item difficulty parameters and the maximum number of attempts, respectively. Generally, an SIRT-MM model outperforms the 2.5PL model in all conditions especially in RMSE when the SIRT-MM model is the true model. Please refer to the Supplementary Material for detailed explanations.

## 4 Empirical analysis

We applied the SIRM-MM model to a real dataset collected from both from college students ( 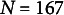 ) and Prolific ( 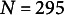 ) participants. They took multiple-choice, multiple-attempt trivia questions about Harry Potter through an online platform, following an AUC procedure. The data was collected between May 2023 to March 2024. There was no missing or incomplete responses. To ensure the quality of data and item parameter estimates, we omitted four test items that were generally very difficult and had very few correct responses at the first attempt. The resulting response matrix included multiple-attempt responses of 462 examinees to 27 test items with four answer options. Because the sample size ( 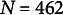 ) was limited, based on the sample size guidelines from our simulation studies, only two candidate SIRT-MM models were fit to the data: (1) the simplest SIRT-MM model without any freely estimated 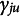 , and (2) an SIRT-MM model with freely estimated 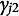 only. We chose a better model with smaller AIC and BIC values between the two candidate models. We also fitted GRM and NRM for comparison. For this analysis, we fixed the maximum number of attempts to two so only data for the first two attempts were used to fit the two candidate models. We compared the 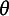 estimates and test information function derived using two attempts against those derived from only the first attempt data. In the Supplementary Material, we present the resulting item parameter estimates (Supplementary Table S5), the histograms of the number of attempts for each item (Supplementary Figure S7) and the item category response functions (Supplementary Figure S8).

Table 6 shows the model fit statistics for the two candidate SIRT-MM models, GRM, and NRM. It shows that both SIRT-MM models lead to smaller AIC and BIC than GRM and NRM in AIC, BIC, and negative log likelihood. AIC is the smallest for the SIRT-MM model with freely estimated 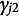 and BIC is the smallest for the simplest SIRT-MM model. As AIC is shown to be more accurate in selecting the correct model in our simulation study when 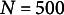 , we selected the SIRT-MM model with freely estimated 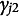 for the subsequent analysis.

**Table 6:** Model-fit statistics for an SIRT-MM model without 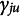 and an SIRT-MM model with a freely estimated 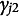

Model	AIC	BIC	Negative log likelihood	Number of parameters
Without 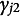	17,866.36	18,089.68	8,879.18	54
With 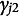	17,778.50	18,113.48	8,808.25	81
GRM	18,896.15	19,231.13	9,367.08	81
NRM	18,832.71	19,279.35	9,308.35	108

**Figure 12:**
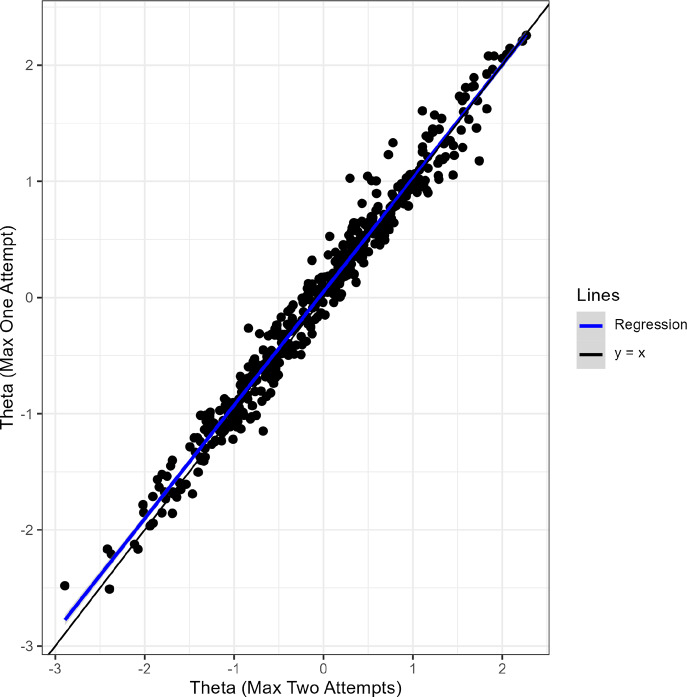
Scatter plot of 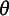 estimated by the SIRT-MM models using only one attempt and two attempts from the real data.

**Figure 13:**
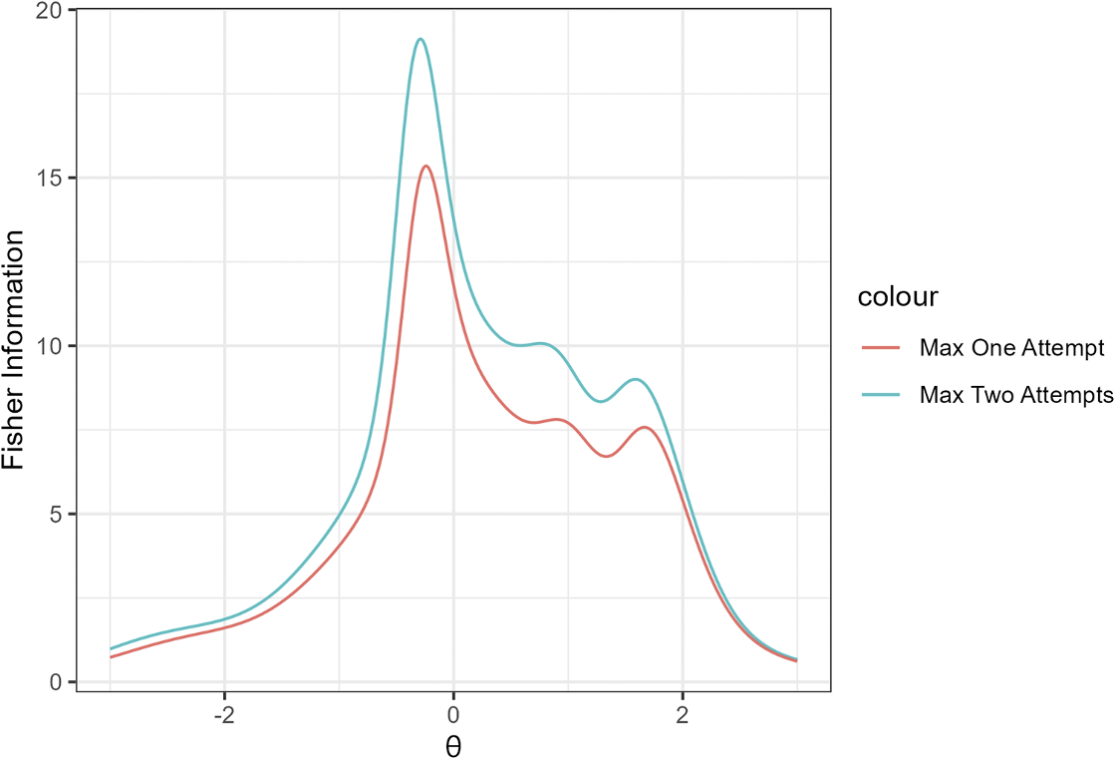
Test information functions of the real data with different maximum numbers of attempts using the item parameter estimates for the SIRT-MM model.

Figure 12 presents the scatter plot of 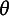 estimated by the SIRT-MM with freely estimated 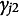 with one- vs. two-attempt data. The result shows that all the 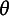 estimates are generally very similar to each other and there is no outlier that yields very different estimates between one or two attempts. However, the precision of these estimates can be different. Figure 13 presents the test information functions derived from the SIRT-MM with one- vs. two-attempt data. There is a consistent increase of test information from one attempt to two attempts, suggesting that allowing two attempts helps gain more information from examinees, which leads to smaller SE of 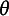 estimates.

## 5 Discussion

This article has proposed and formally derived a family of new sequential item response models (SIRT-MM models) for multiple-choice, multiple-attempt test items that considers the guessing of multiple-choice test items, and the homogeneity and heterogeneity of distractors. We demonstrated that an SIRT-MM model can be used to glean more information from multiple-choice, multiple-attempt items and to provide better scoring, especially for in the region of smaller 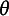 .

Our simulation study included model selection, and item and person parameter recovery. For model selection, we showed that AIC and BIC never selected GRM or NRM when data were generated from SIRT-MM models, demonstrating the unique utility of the SIRT-MM models to model multiple-attempt data. For item parameter recovery, we showed that our implementation of MMLE could recover item parameters very well with 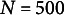 for the simplest SIRT-MM model and with 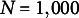 or 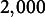 for more complex SIRT-MM models with reasonable test lengths. For person parameter recovery, we showed that an SIRT-MM model consistently outperforms the 2.5PL model in all conditions when former is the true model. Also, the person parameter recovery results suggested that 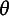 can be estimated reasonably well even with a small sample size. Taken together, one could consider adopting a multiple-attempt procedure and SIRT-MM models to improve measurement precision.

One limitation of this study is that we have not fully investigated different possible parameterizations of SIRT-MM models. First, our proposed models do not allow a freely estimated pseudo-guessing parameter for each item. By doing so, SIRT-MM models could be compared against the actual 3PL model, instead of the 2.5PL model. That being said, it is worth noting that a previous study showed that fixing the pseudo-guessing parameter in 3PL model provides a stable and accurate item estimation solution (Han, 2012), and thus our study still provides practical utility. Second, our proposed models do not focus on varying the 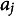 parameter at each attempt. The concept of allowing the item discrimination parameter to change at each attempt by introducing 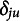 parameters is explained and discussed in the Supplementary Material. This extension could be important as item discrimination in a traditional SIRT model could decrease with each attempt (Lyu et al., 2023). Both extensions imply estimating many additional item parameters, which could cause convergence issues or inaccurate item parameters unless we have a huge sample size. Future work could consider regularization for item parameter estimation or Bayesian estimation to help accurately estimate more item parameters even with a smaller sample size.

A few additional limitations should be noted about the current study. First, our simulation did not evaluate all the combinations of simulation conditions for item parameters. The sample size requirement would be different depending on various factors including the complexity of a model and the distributions of true parameters including 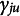 . As suggested by a reviewer, interactions between such factors should also be evaluated. In addition, our models did not model learning or growth in this context. For future work, we can consider a use case where growth-curve SIRT-MM models similar to Culpepper (2014) could be formulated and used to track examinees’ learning.

## Supporting information

Lu et al. supplementary material Lu et al. supplementary material

## Footnotes

1 One approach to model  is to model  directly as a function of item parameters and  . However, an issue of this parameterization is that  will depend on attempt-specific parameters from later attempts, which makes it not an SIRT model anymore. Also, we cannot estimate the model when we do not have a large sample size or we set the maximum number of attempts to be less than *K* where later attempts are not available.

## Supplementary material.

The supplementary material for this article can be found at https://doi.org/10.1017/psy.2024.18. The R package that includes the estimation program and sample real data are available at https://github.com/luyikei/sirtmm.
