# Supplementary material for: A Family of Sequential Item Response Models for Multiple-Choice, Multiple-Attempt Test Items
Source: Psychometrika. 2025 Jan 3;90(1):331–57. doi: 10.1017/psy.2024.18 (PMC12478618; doi:10.1017/psy.2024.18)
Supplement: Lu et al. supplementary material [file S0033312324000188sup001.pdf]

Supplementary Materials

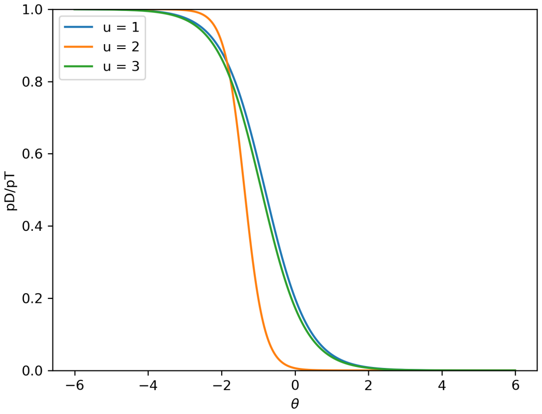

Figure S1: $\frac{\overline{p_D}(\theta)}{p_T(\theta)}$ when $a = 1.7, b = 0.0, \gamma_2 = 1, \gamma_3 = 0.1, \delta_2 = 2, \delta_3 = 0, K = 4$

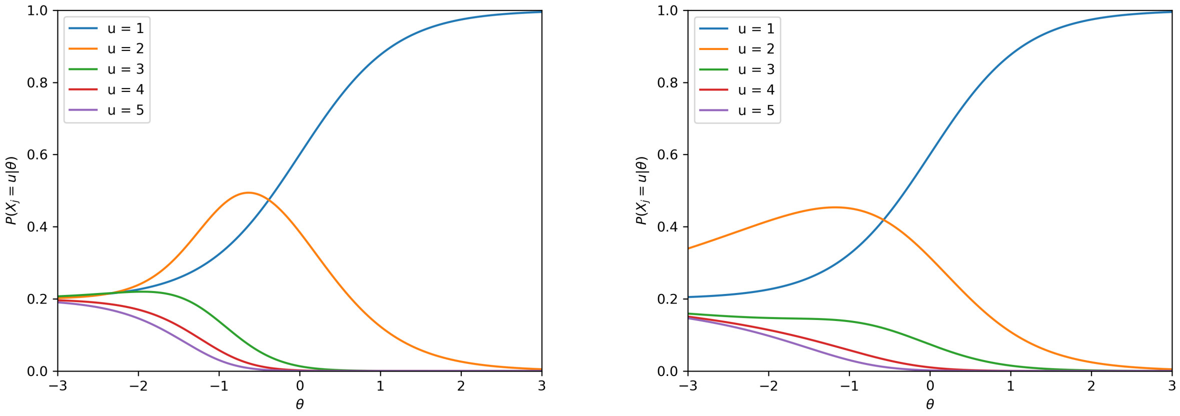

(a) $\delta_2 = 1$ (b) $\delta_2 = -1$

Figure S2: Item category response function: Item category response function: $a = 1.7, b = 0.0, \gamma_2 = 1, \gamma_3 = 0.5, \gamma_4 = 0, \delta_3 = 0, \delta_4 = 0, K = 5$ with different δ_2

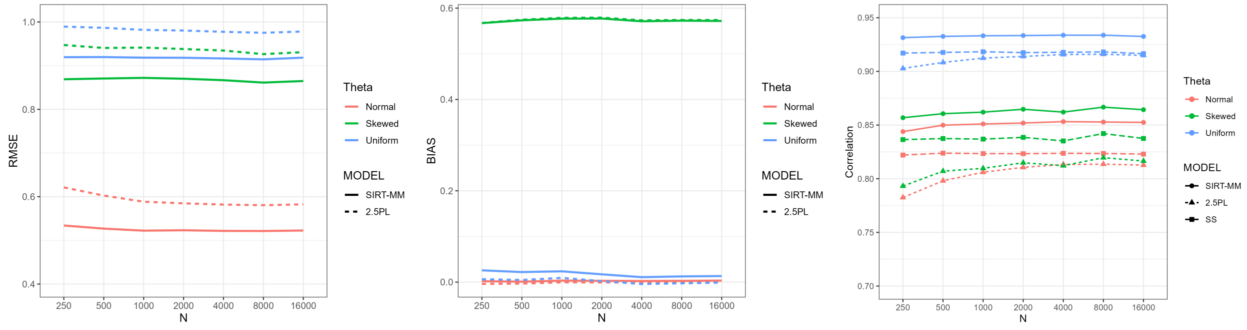

(a) RMSE (b) Bias (c) Correlation

Figure S3: Person parameter statistics when $M = 20$, $a_j \sim \text{Unif}(0.75, 1.33)$, $b_j \sim \text{Unif}(-2, 2)$, and $\gamma_{j2} \sim \text{Unif}(-1, 1)$ varying θ. M is the number of items administered. The scoring scheme used in classical test theory is denoted as SS in the correlation plot.

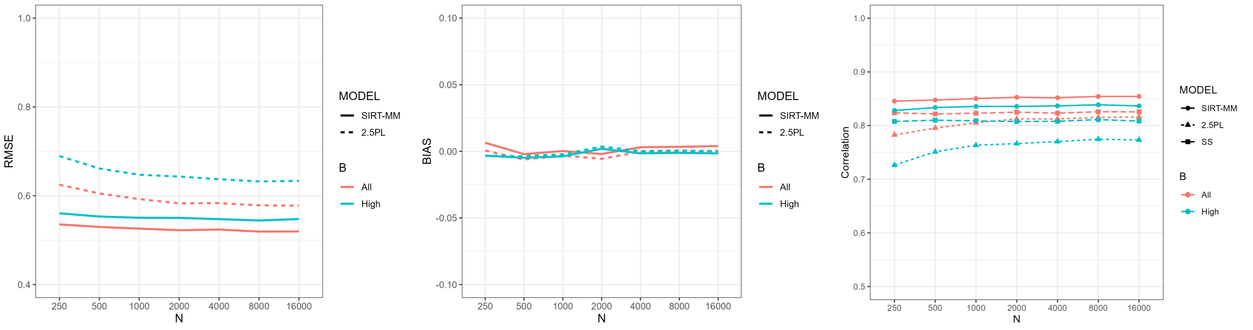

(a) RMSE (b) Bias (c) Correlation

Figure S4: Person parameter statistics when $M = 20$, $\theta \sim N(0, 1)$, $a_j \sim \text{Unif}(0.75, 1.33)$, and $\gamma_{j2} \sim \text{Unif}(-1, 1)$ varying b_j. M is the number of items administered. The scoring scheme used in classical test theory is denoted as SS in the correlation plot.

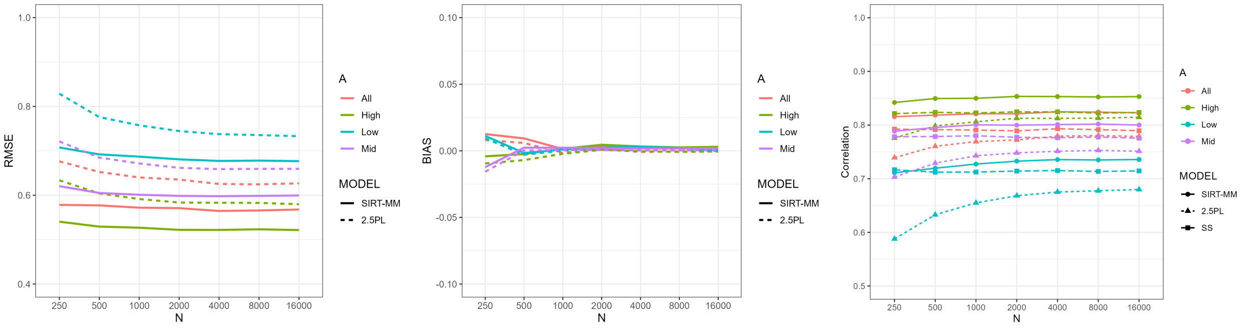

(a) RMSE (b) Bias (c) Correlation

Figure S5: Person parameter statistics when $M = 20$, $\theta \sim N(0, 1)$, $b_j \sim \text{Unif}(-2, 2)$, and $\gamma_{j2} \sim \text{Unif}(-1, 1)$ varying a_j. M is the number of items administered. The scoring scheme used in classical test theory is denoted as SS in the correlation plot.

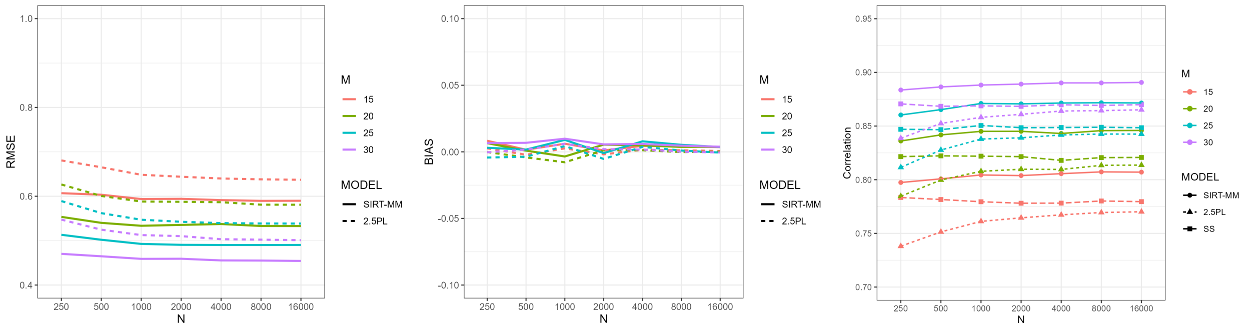

(a) RMSE (b) Bias (c) Correlation

Figure S6: Person parameter statistics when the maximum number of attempts is two, $\theta \sim N(0,1), a \sim \text{Unif}(0.75, 1.33), b \sim \text{Unif}(-2, 2)$, and $\gamma_{j2} \sim \text{Unif}(-1, 1)$. M is the number of items administered. The scoring scheme used in classical test theory is denoted as SS in the correlation plot.

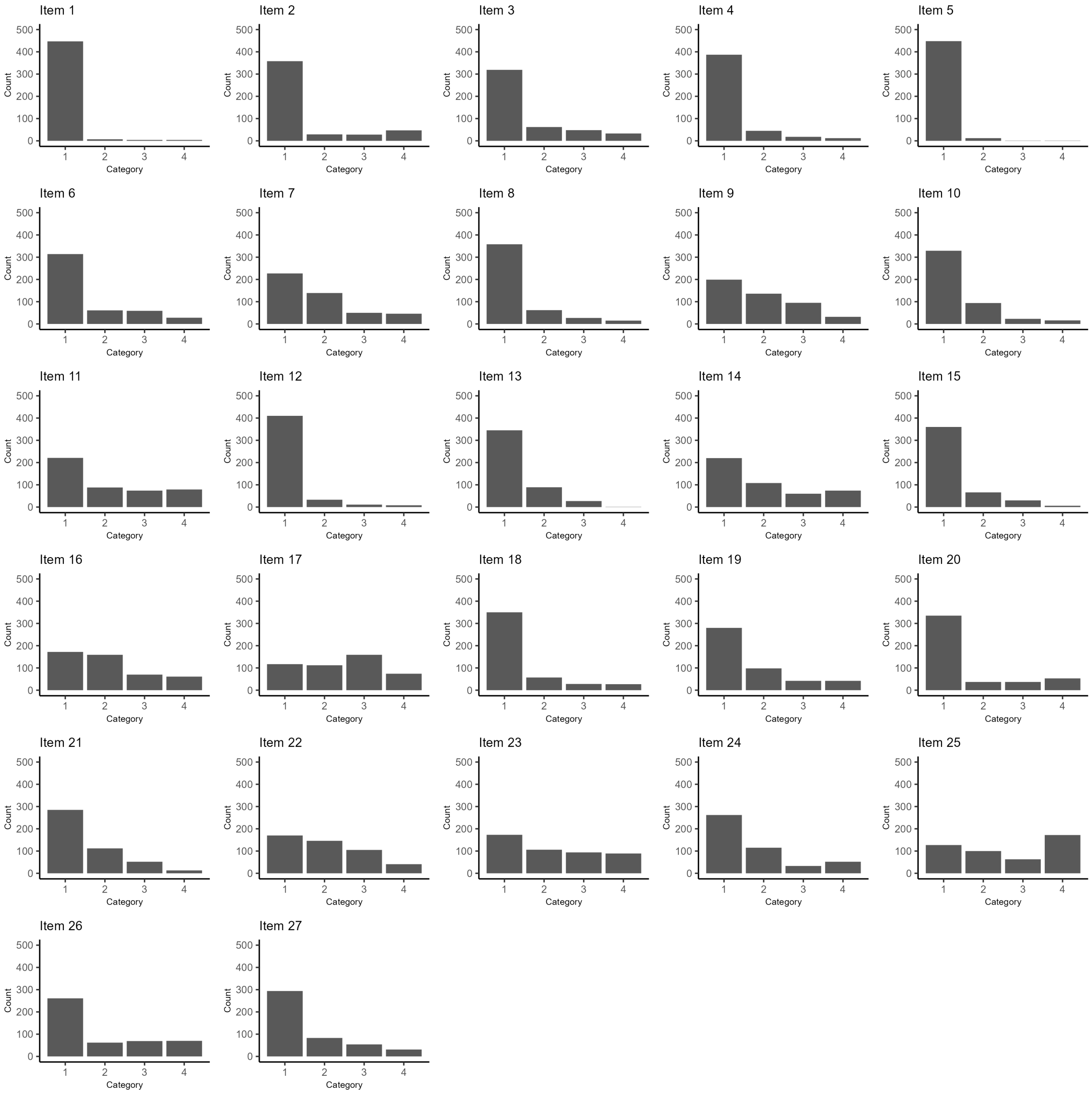

Figure S7: Bar plots of the number of attempts for the real items

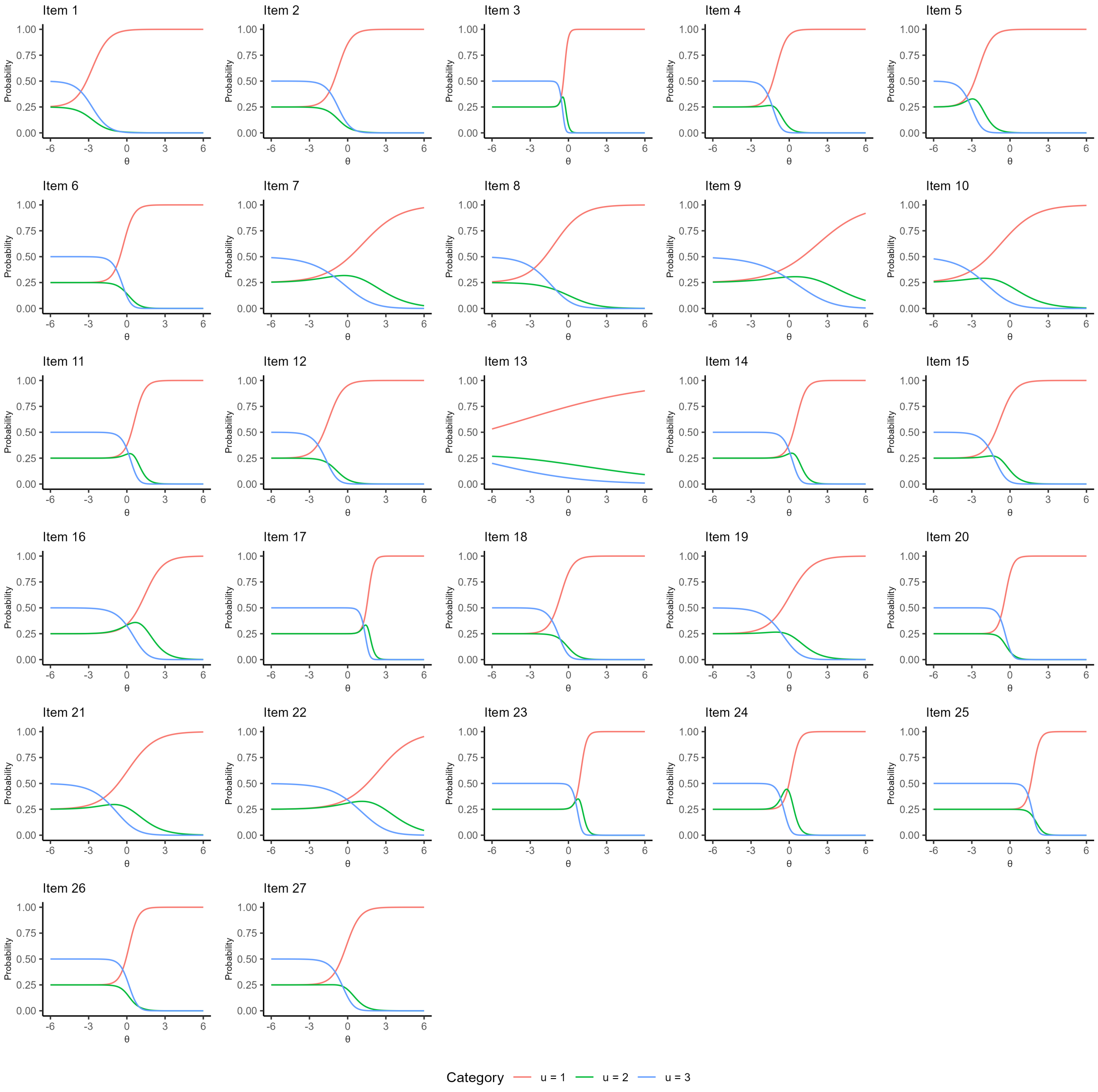

Figure S8: Estimated item category response functions for the real items

Table S1: Item recovery statistics for different θ distributions

		SE			BIAS			RMSE			CONV
θ	N	b_j	a_j	γ_{j2}	b_j	a_j	γ_{j2}	b_j	a_j	γ_{j2}	
Normal	250	0.32	0.24	0.53	-0.00	0.04	-0.01	0.41	0.32	0.79	0.98
	500	0.20	0.16	0.35	0.01	0.01	-0.00	0.27	0.21	0.40	1.00
	1000	0.13	0.11	0.24	0.01	0.00	-0.01	0.17	0.15	0.26	1.00
	2000	0.09	0.08	0.17	0.01	-0.00	0.00	0.11	0.10	0.17	1.00
	4000	0.07	0.06	0.12	0.01	-0.00	-0.00	0.08	0.07	0.13	1.00
	8000	0.05	0.04	0.08	0.01	-0.01	-0.00	0.06	0.05	0.09	1.00
	16000	0.03	0.03	0.06	0.01	-0.00	0.00	0.04	0.04	0.06	1.00
Skewed	250	0.37	0.27	0.48	0.61	0.19	-0.06	0.83	0.43	0.74	0.99
	500	0.25	0.18	0.32	0.59	0.15	-0.01	0.73	0.32	0.39	1.00
	1000	0.14	0.13	0.23	0.57	0.13	-0.01	0.62	0.25	0.28	1.00
	2000	0.10	0.09	0.15	0.55	0.14	-0.01	0.58	0.23	0.20	1.00
	4000	0.07	0.06	0.11	0.55	0.12	-0.00	0.58	0.21	0.15	1.00
	8000	0.05	0.04	0.07	0.55	0.13	-0.00	0.57	0.20	0.13	1.00
	16000	0.03	0.03	0.05	0.55	0.13	-0.01	0.57	0.20	0.12	1.00
Uniform	250	0.13	0.34	0.28	0.04	0.92	-0.01	0.54	1.03	0.40	1.00
	500	0.09	0.24	0.19	0.02	0.88	-0.01	0.52	0.95	0.33	1.00
	1000	0.06	0.16	0.14	0.02	0.86	-0.00	0.52	0.90	0.29	1.00
	2000	0.05	0.11	0.10	0.01	0.85	0.02	0.52	0.88	0.27	1.00
	4000	0.03	0.08	0.07	0.01	0.84	-0.00	0.52	0.86	0.26	1.00
	8000	0.02	0.06	0.05	0.01	0.84	-0.00	0.51	0.86	0.26	1.00
	16000	0.02	0.04	0.03	0.01	0.83	0.01	0.52	0.85	0.25	1.00

Table S2: Item recovery statistics for different a parameters

		SE			BIAS			RMSE			CONV
a	N	b_j	a_j	γ_{j2}	b_j	a_j	γ_{j2}	b_j	a_j	γ_{j2}	
Low, Unif$(0.44, 0.75)$	250	1.20	0.18	1.23	0.01	0.03	-0.03	1.36	0.27	1.22	0.91
	500	0.37	0.12	0.55	-0.01	0.01	0.00	0.48	0.17	0.60	0.99
	1000	0.23	0.09	0.35	0.01	0.01	0.00	0.31	0.12	0.37	1.00
	2000	0.15	0.06	0.24	0.00	0.00	-0.00	0.20	0.08	0.24	1.00
	4000	0.10	0.04	0.17	0.00	0.00	0.00	0.14	0.06	0.18	1.00
	8000	0.07	0.03	0.12	0.00	-0.00	-0.00	0.09	0.04	0.12	1.00
	16000	0.05	0.02	0.08	0.00	-0.00	0.00	0.06	0.03	0.09	1.00
Mid, Unif$(0.58, 0.98)$	250	0.53	0.20	0.73	-0.04	0.03	-0.02	0.61	0.27	0.75	0.99
	500	0.26	0.14	0.42	0.01	0.02	-0.01	0.34	0.18	0.44	1.00
	1000	0.17	0.10	0.28	0.00	0.01	0.01	0.21	0.13	0.30	1.00
	2000	0.12	0.07	0.20	0.00	-0.00	0.00	0.15	0.09	0.20	1.00
	4000	0.08	0.05	0.14	0.00	-0.00	0.00	0.10	0.06	0.15	1.00
	8000	0.06	0.03	0.10	0.00	-0.00	-0.00	0.07	0.04	0.10	1.00
	16000	0.04	0.02	0.07	0.00	-0.00	-0.00	0.05	0.03	0.07	1.00
High, Unif$(0.75, 1.33)$	250	0.32	0.23	0.53	-0.01	0.02	0.04	0.42	0.32	0.84	0.99
	500	0.19	0.16	0.34	0.00	0.02	0.01	0.23	0.21	0.35	1.00
	1000	0.13	0.11	0.24	0.00	0.01	-0.00	0.16	0.15	0.26	1.00
	2000	0.09	0.08	0.17	0.00	0.00	0.00	0.12	0.11	0.17	1.00
	4000	0.06	0.06	0.12	0.00	-0.00	-0.01	0.08	0.07	0.12	1.00
	8000	0.05	0.04	0.08	0.00	-0.00	-0.00	0.06	0.05	0.09	1.00
	16000	0.03	0.03	0.06	0.00	-0.00	-0.00	0.04	0.04	0.06	1.00
All, Unif$(0.44, 1.33)$	250	0.44	0.21	0.65	-0.00	0.03	-0.02	0.56	0.30	0.79	0.98
	500	0.25	0.15	0.41	0.01	0.02	0.02	0.32	0.20	0.52	1.00
	1000	0.17	0.10	0.28	0.01	0.01	0.01	0.21	0.14	0.29	1.00
	2000	0.11	0.07	0.19	0.00	0.00	0.00	0.14	0.10	0.21	1.00
	4000	0.08	0.05	0.13	0.00	-0.00	0.00	0.10	0.07	0.14	1.00
	8000	0.06	0.04	0.10	0.01	-0.00	-0.01	0.07	0.05	0.10	1.00
	16000	0.04	0.03	0.07	0.00	-0.00	-0.00	0.05	0.03	0.07	1.00

Table S3: Item recovery statistics for different b parameters

		SE			BIAS			RMSE			CONV
b	N	b_j	a_j	γ_{j2}	b_j	a_j	γ_{j2}	b_j	a_j	γ_{j2}	
All, Unif$(-2, 2)$	250	0.33	0.24	0.55	0.03	0.05	0.03	0.45	0.33	1.00	1.00
	500	0.20	0.16	0.36	0.01	0.00	-0.00	0.25	0.22	0.39	1.00
	1000	0.13	0.11	0.24	0.00	0.00	-0.02	0.16	0.14	0.26	1.00
	2000	0.09	0.08	0.17	-0.00	0.00	-0.00	0.11	0.10	0.17	1.00
	4000	0.07	0.06	0.12	0.01	0.00	-0.00	0.08	0.07	0.13	1.00
	8000	0.05	0.04	0.08	0.00	-0.01	-0.00	0.06	0.05	0.09	1.00
	16000	0.03	0.03	0.06	0.00	-0.00	-0.00	0.04	0.04	0.06	1.00
High, Unif$(0, 2)$	250	0.31	0.25	0.49	0.05	0.06	-0.07	0.40	0.35	0.73	1.00
	500	0.19	0.17	0.32	0.02	0.02	-0.01	0.25	0.22	0.34	1.00
	1000	0.13	0.12	0.22	0.01	0.01	-0.00	0.17	0.16	0.23	1.00
	2000	0.09	0.08	0.15	0.01	0.00	-0.00	0.12	0.11	0.16	1.00
	4000	0.07	0.06	0.11	0.00	-0.00	-0.00	0.08	0.07	0.12	1.00
	8000	0.05	0.04	0.08	0.00	-0.00	-0.00	0.06	0.05	0.08	1.00
	16000	0.03	0.03	0.05	-0.00	-0.00	-0.00	0.04	0.04	0.06	1.00

Table S4: Item recovery statistics when the maximum number of attempts is two

		SE			BIAS			RMSE			CONV
b	N	b_j	a_j	γ_{j2}	b_j	a_j	γ_{j2}	b_j	a_j	γ_{j2}	
15	250	0.35	0.25	0.55	0.04	0.07	0.04	0.48	0.40	0.88	1.00
	500	0.21	0.17	0.35	0.01	0.02	-0.01	0.27	0.25	0.39	1.00
	1000	0.14	0.12	0.24	-0.01	0.01	-0.00	0.18	0.17	0.27	1.00
	2000	0.10	0.08	0.17	0.00	-0.00	-0.00	0.12	0.12	0.19	1.00
	4000	0.07	0.06	0.12	0.01	-0.00	-0.01	0.09	0.08	0.13	1.00
	8000	0.05	0.04	0.08	0.00	-0.01	-0.00	0.06	0.06	0.09	1.00
	16000	0.03	0.03	0.06	0.00	-0.00	0.00	0.04	0.04	0.06	1.00
20	250	0.36	0.26	0.54	0.03	0.07	-0.01	0.47	0.39	0.99	0.97
	500	0.20	0.17	0.35	-0.00	0.03	-0.01	0.26	0.24	0.42	1.00
	1000	0.14	0.12	0.24	0.00	0.01	-0.00	0.18	0.16	0.26	1.00
	2000	0.10	0.08	0.17	0.01	-0.00	0.00	0.12	0.11	0.18	1.00
	4000	0.07	0.06	0.12	0.01	-0.01	-0.00	0.08	0.08	0.13	1.00
	8000	0.05	0.04	0.08	0.00	-0.00	-0.00	0.06	0.05	0.09	1.00
	16000	0.03	0.03	0.06	0.01	-0.01	-0.00	0.04	0.04	0.06	1.00
25	250	0.32	0.25	0.53	0.01	0.05	0.03	0.42	0.36	0.84	0.99
	500	0.20	0.17	0.35	0.00	0.02	0.01	0.25	0.23	0.47	1.00
	1000	0.14	0.12	0.25	-0.00	0.00	-0.01	0.18	0.16	0.27	1.00
	2000	0.10	0.08	0.17	0.01	-0.00	-0.01	0.11	0.11	0.18	1.00
	4000	0.07	0.06	0.12	0.01	-0.00	-0.00	0.08	0.07	0.12	1.00
	8000	0.05	0.04	0.08	0.00	-0.00	0.00	0.06	0.05	0.09	1.00
	16000	0.03	0.03	0.06	0.01	-0.00	-0.00	0.04	0.04	0.06	1.00
30	250	0.32	0.25	0.52	0.01	0.04	0.06	0.40	0.34	1.08	0.98
	500	0.20	0.17	0.35	0.01	0.01	-0.00	0.25	0.22	0.44	1.00
	1000	0.14	0.12	0.24	0.02	-0.00	-0.01	0.17	0.15	0.27	1.00
	2000	0.10	0.08	0.17	0.00	-0.00	0.00	0.11	0.10	0.18	1.00
	4000	0.07	0.06	0.12	0.01	-0.00	0.00	0.08	0.07	0.13	1.00
	8000	0.05	0.04	0.08	0.00	-0.01	0.00	0.06	0.05	0.09	1.00
	16000	0.03	0.03	0.06	0.01	-0.01	-0.00	0.04	0.04	0.06	1.00

Table S5: Item parameter estimates for the real items (standard error estimates are in parentheses)

Item	b_j	a_j	γ_{j2}
1	-2.71 (0.40)	1.54 (0.34)	-3.06 (1.13)
2	-0.74 (0.10)	1.99 (0.27)	-2.15 (0.74)
3	-0.30 (0.05)	7.02 (1.10)	0.07 (0.10)
4	-1.01 (0.10)	2.47 (0.34)	-0.22 (0.21)
5	-2.44 (0.29)	1.98 (0.42)	0.15 (0.58)
6	-0.26 (0.08)	2.44 (0.32)	-0.68 (0.23)
7	1.19 (0.27)	0.68 (0.14)	0.26 (0.38)
8	-1.09 (0.20)	0.93 (0.15)	-1.61 (0.47)
9	2.26 (0.57)	0.57 (0.15)	0.08 (0.52)
10	-0.72 (0.21)	0.74 (0.13)	-0.20 (0.40)
11	0.62 (0.08)	2.57 (0.38)	-0.05 (0.16)
12	-1.48 (0.16)	1.78 (0.26)	-0.68 (0.33)
13	-3.44 (1.95)	0.20 (0.11)	-1.80 (1.78)
14	0.57 (0.07)	2.64 (0.38)	-0.02 (0.15)
15	-0.77 (0.11)	1.73 (0.22)	-0.25 (0.23)
16	1.42 (0.17)	1.40 (0.24)	0.45 (0.23)
17	1.62 (0.09)	4.74 (1.35)	0.08 (0.18)
18	-0.60 (0.09)	2.15 (0.28)	-0.60 (0.23)
19	0.08 (0.12)	1.15 (0.17)	-0.47 (0.28)
20	-0.35 (0.06)	3.46 (0.48)	-0.88 (0.29)
21	0.07 (0.14)	0.93 (0.15)	-0.09 (0.30)
22	2.45 (0.52)	0.76 (0.20)	0.37 (0.45)
23	0.99 (0.06)	4.49 (0.82)	0.12 (0.12)
24	0.19 (0.06)	3.37 (0.43)	0.46 (0.11)
25	1.81 (0.13)	3.78 (1.34)	-0.44 (0.43)
26	0.18 (0.07)	2.77 (0.40)	-1.28 (0.48)
27	-0.11 (0.09)	1.84 (0.24)	-0.47 (0.22)

Changing Item Discrimination at Different Attempts

Furthermore, even more complex types of item category response functions can be modeled. As Lyu, Bolt, and Westby (2023) pointed out, when ignoring the influence of the item-specific factor of an item, we expect to see reduced item discrimination at later attempts. We propose to introduce another attempt-specific parameter $\delta_u \in \mathbb{R}$ for $u = 2 \ldots K-1$ while setting $\delta_1 = \delta_K \equiv 0$ to take into account reduced item discrimination at later attempts:

$$\frac{\overline{p_D}(\theta, u)}{p_T(\theta, u)} = \frac{1}{1 + K\exp((a+\delta_u)(\theta - b + \gamma_u))}. \tag{1}$$

Therefore,

$$\begin{aligned}\frac{1}{H(\theta, u)} &= 1 + (K-u)\frac{\overline{p_D}(\theta, u)}{p_T(\theta, u)} \\ &= 1 + \frac{K-u}{1 + K\exp((a+\delta_u)(\theta - b + \gamma_u))}.\end{aligned} \tag{2}$$

This leads to:

$$\begin{aligned}P(X = u|\theta) &= H(\theta, u)\prod_{k=1}^{u-1}[1 - H(\theta, k)] \\ &= \frac{(K-1)![1 + K\exp((a+\delta_u)(\theta - b + \gamma_u))]}{(K-u)!\prod_{k=1}^{u}[K - k + 1 + K\exp((j+\delta_k)(\theta - b + \gamma_k))]}.\end{aligned} \tag{3}$$

========================

Insert Figure S1 about here

========================

δ parameters will be able to introduce a type of an "interaction" effect. Specifically, we will be able to vary whether $\frac{\overline{p_D}(\theta,u)}{p_T(\theta,u)}$ increases or decreases as u increases depending on θ ranges. Figure S1 shows example functions of $\frac{\overline{p_D}(\theta,u)}{p_T(\theta,u)}$ when $a = 1.7$, $b = 0.0$, $K = 4$, $\gamma_2 = 1$, $\gamma_3 = 0.1$, $\delta_2 = 2$, and $\delta_3 = 0$. Note that $\frac{\overline{p_D}(\theta,u)}{p_T(\theta,u)}$ at $u = 1$ and $u = 2$ have an intersection around $\theta = -1.5$, and $\frac{\overline{p_D}(\theta,u)}{p_T(\theta,u)}$ will increase faster when $u = 2$ for lower θ while $\frac{\overline{p_D}(\theta,u)}{p_T(\theta,u)}$ will decrease faster at $u = 2$ for higher θ.

We can interpret δ parameters in a similar way to how we interpret γ parameters. However, it requires a plot of $\frac{\overline{p_D}(\theta,u)}{p_T(\theta,u)}$ to identify how $\frac{\overline{p_D}(\theta,u)}{p_T(\theta,u)}$ will change as u increases. We need to interpret δ conditioning on specific θ values because its effect is not uniform across all θ values.

==========================

Insert Figure S2 about here

==========================

Figure S2 shows example ICRFs of

$a = 1.7, b = 0.0, \gamma_2 = 1, \gamma_3 = 0.5, \gamma_4 = 0, \delta_3 = 0, \delta_4 = 0, K = 5$ and different δ_2s. The left panel shows the ICRFs when $\delta_2 = 1$ and the right panel shows the ICRFs when $\delta_2 = -1$. When we compare between the two panels, the ICRFs for $u = 2$ have different shapes. Specifically, when $\delta_2 = -1$, the ICRF for $u = 2$ has a flatter top than $\delta_2 = 1$. Therefore, by introducing δ parameters, we can model more different types of item response category functions.

Other Descriptions

Figure S3 shows the person recovery statistics varying θ distributions when $M = 20, a_j \sim \text{Unif}(0.75, 1.33), b_j \sim \text{Unif}(-2, 2)$, and $\gamma_{j2} \sim \text{Unif}(-1, 1)$. Again, we find that the SIRT-MM consistently outperforms 2.5PL in RMSE and correlations. In particular, we find that the SIRT-MM especially outperforms 2.5PL when the true θ distribution is positively skewed, as shown by the large reduction in RMSE and the greatest increment in correlation compared to other models. This again supports our finding that the SIRT-MM can recover item information in lower θ very well thanks to its ability to glean partial (mis)information manifested in response patterns.

Figure S4 shows the person recovery statistics varying b ranges when $M = 20$, $\theta \sim N(0, 1)$, $a_j \sim \text{Unif}(0.75, 1.33)$, and $\gamma_{j2} \sim \text{Unif}(-1, 1)$. In these conditions, we compare the ordinary condition, $b \sim \text{Unif}(-2, 2)$ against the “difficult item” condition, $b \sim \text{Unif}(0, 2)$. As expected, even when test items are relatively difficult, the SIRT-MM model can still perform comparably well by recovering item information in lower θ. The difference between these two b_j conditions for the SIRT-MM is smaller in all the metrics whereas is relatively larger for the 2.5PL model. For both the SIRT-MM model and the 2.5 PL model, having a skewed θ distribution has a larger effect on person parameter recovery than a limited range of b. This is because marginal maximum likelihood estimation for item parameter estimation assumes a standard normal for θ distribution, and thus having a different population distribution leads to wrong item parameter estimates.

Figure S5 shows the person recovery statistics varying a ranges when $M = 20$, $\theta \sim N(0, 1)$, $b_j \sim \text{Unif}(-2, 2)$, and $\gamma_{j2} \sim \text{Unif}(-1, 1)$. Across a_j ranges, the SIRT-MM model consistently

outperforms the 2.5PL model in recovering θ, as demonstrated by smaller RMSE and larger correlations.

Figure S6 shows the person recovery statistics when the maximum number of attempts is two, $\theta \sim N(0, 1)$, $a_j \sim \text{Unif}(0.75, 1.33)$, $b_j \sim \text{Unif}(-2, 2)$, and $\gamma_{j2} \sim \text{Unif}(-1, 1)$. The results are very similar to Figure **??**, which can be explained by the fact that the majority of the population will not reach the third attempt since θ is sampled from the standard normal distribution. This result suggests that AUC might not necessarily be needed to gain useful information from examinees; instead, setting the maximum number of attempts for multiple attempts might be beneficial in reducing the burden of examinees while preserving the maximum efficiency of the multiple-attempts format. However, AUC might still be the most effective in cases such as low-ability population or a large γ_{j3}. Therefore, we recommend identifying these possible factors before deciding to limit the number of attempts.

Table S1 shows the item recovery statistics varying θ distributions when $M = 20$, $a_j \sim \text{Unif}(0.75, 1.33)$, $b_j \sim \text{Unif}(-2, 2)$, and $\gamma_{j2} \sim \text{Unif}(-1, 1)$. For the skewed θ distribution, b_j parameters are positively biased by around 0.55, and for the uniform θ distribution, a_j parameters are positively biased by around 0.83. The RMSE for all parameters could be significantly inflated if the θ distribution does not follow the standard normal distribution.

Table S2 shows the item recovery statistics varying a_j parameters when $M = 20$, $\theta \sim N(0, 1)$, $b_j \sim \text{Unif}(-2, 2)$, and $\gamma_{j2} \sim \text{Unif}(-1, 1)$. We notice the same pattern in Table 3 that the quality of item parameter estimates improves as N gets larger. In addition, we can observe that standard errors and RMSE are larger when true a_j is smaller. Especially, for the low a_j condition, we need at least $N \geq 500$ to have reliable item parameter estimates since b_j parameters are estimated

poorly, which is demonstrated by the standard error of 1.20, and the RMSE of 1.36.

Table S3 shows the item recovery statistics varying b_j parameters when $M = 25$, $\theta \sim N(0, 1)$, $a_j \sim \text{Unif}(0.75, 1.33)$, and $\gamma_{j2} \sim \text{Unif}(-1, 1)$. Again, the quality of item parameter estimates improves as N gets larger. Contrary to how ranges of a_j parameters could affect item parameter estimation, limiting b_j parameters to a high range does not seem to affect item parameter estimation much. This is partially due to the fact that SIRT-MM models can recover item information from relatively lower θ population since they are expected to respond with more attempts, which generates more information available for estimation.

Table S4 shows the item recovery statistics when the maximum number of attempts is two, $\theta \sim N(0, 1)$, $a_j \sim \text{Unif}(0.75, 1.33)$, $b_j \sim \text{Unif}(-2, 2)$, and $\gamma_{j2} \sim \text{Unif}(-1, 1)$. Note that the results are very similar to Table 4, which has the maximum number of attempts of four (three and four are technically the same). In fact, many values of RMSE are better with the maximum number of attempts of two. This could be because the estimation algorithm might become more stable when the number of response categories is smaller (in this case, three). Therefore, we recommend setting the maximum number of attempts when there are many examinees who stop responding with more attempts or missing values.

References

Lyu, W., Bolt, D. M., & Westby, S. (2023, June). Exploring the Effects of Item-Specific Factors in Sequential and IRTree Models. *Psychometrika*. doi: 10.1007/s11336-023-09912-x
